# Fucoidan-decorated metal-zoledronic acid nanocomplexes suppress tumor metastasis by inducing ferroptotic cell death and enhancing cancer immunotherapy

**DOI:** 10.1186/s12951-025-03473-0

**Published:** 2025-06-02

**Authors:** Hsin-Ting Tsai, Chi Lin, Chu-Hung Chung, Wen-Jing Hsu, Ming-Yi Hsieh, Ming-Cheng Chiang, Tzu-Wei Lu, Fwu-Long Mi, Cheng-Wei Lin

**Affiliations:** 1 Department of Biochemistry and Molecular Cell Biology, School of Medicine, College of Medicine, Taipei Medical University, 250 Wu-Xing Street, Taipei, 11031 Taiwan; 2 Graduate Institute of Medical Sciences, College of Medicine, Taipei Medical University, Taipei, 11031 Taiwan; 3 Graduate Institute of Nanomedicine and Medical Engineering, College of Medical Bioengineering, Taipei Medical University, Taipei, 11031 Taiwan; 4 Department of Biomedical Science and Environmental Biology, Kaohsiung Medical University, Kaohsiung, 807378 Taiwan

**Keywords:** Fucoidan, Ferroptosis, Chemoimmunotherapy, Supramolecule, Zoledronic acid

## Abstract

Metastatic tumor cells that escape from immune surveillance are a dilemma in cancer treatment, and thus developing selective targeting agents to treat metastatic tumor and reinstate immune perception is imperative for clinical applications. Herein, a multifunctional nanoplatform of supramolecular assembled nanoparticles (SANs) comprising a core structure of metal ion (Fe^3+^) and organic ligands including tannic acid (TA), and zoledronic acid (Zol) was developed. The FTZ SANs was further decorated with fucoidan (Fu), a P-selectin ligand, which greatly enhanced specific binding affinity of FTZ@Fu SANs towards metastatic tumor cells and suppressed tumor aggressiveness. The Fe-TA-Zol coordination network constructed through competitive ligand substitution facilitated the releases of Zol in response to the acidic tumor microenvironment (TME), which also benefited iron redox cycling of the Fenton reaction and further trigger ferritinophagy. Subsequently, Zol coordinately exerted ferroptotic-inducing activity accompanied by induction of stimulator of interferon genes (STING) pathway to aggravate immunogenic cell death (ICD) and enhance the antitumor immune response. Furthermore, FTZ@Fu effectively attenuated the immunosuppressive TME to suppress tumor growth and distant metastasis, and FTZ@Fu potentiated the therapeutic efficacy in combination with immune checkpoint blockade (ICB) therapy. Importantly, FTZ@Fu SANs suppressed metastatic tumor growth and reshaped the immune microenvironment. Our nanosystem provides a promising avenue for synergetic cancer targeting and chemoimmunotherapy, paving the way for targeted therapeutic strategies.

**Graphical abstract:**

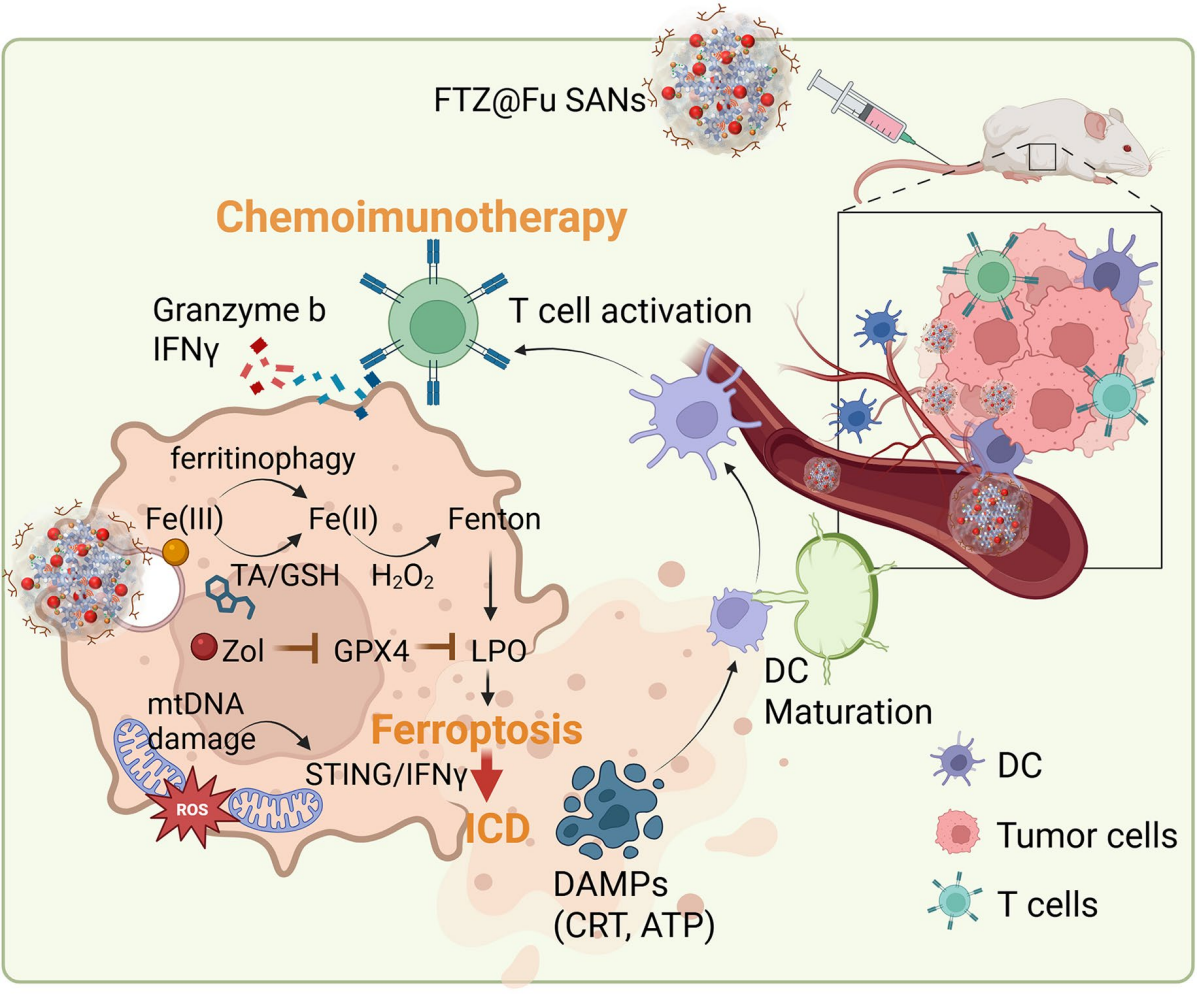

**Supplementary Information:**

The online version contains supplementary material available at 10.1186/s12951-025-03473-0.

## Introduction

Metastasis is responsible for 90% of cancer-related mortalities, and overcoming tumor metastasis is one of the top challenges in cancer treatment. Tumor dissemination is a complex and multistep process, requiring cancer cells to overcome influences of multiple obstructions and stresses such as anoikis resistance, collective migration in the bloodstream, survival in different tumor microenvironments (TMEs), and foremost, escape of immune surveillance in different growing sites. The reprogramming of metabolism allows tumor cells to thrive in such arduous conditions and metastatic sites which are hypoxic, with limited nutrients, and with abundant metabolic stresses, and metabolic alterations enable tumor cells to mitigate lipid peroxidation [1–3]. It is unequivocally conceivable that an increase in glycolysis promotes tumor growth, invasiveness, and resistance to drug treatment [4–6]. In addition, the accumulation of lactate, which is the end metabolite of glycolysis, promotes acidification of the TME and immune suppression. Moreover, increases in components of the antioxidant system such as glutathione (GSH) in tumor cells render the ability to combat various oxidative stresses. In this context, the induction of ferroptosis, a newly discovered form of ion-dependent cell death by dysregulation of metabolic processes, has gained much attention in cancer treatment [7]. Ferroptosis is characterized by the culmination of overwhelming lipid peroxidation (LPO), which is triggered by excess ions to catalyze Fenton or Fenton-like reactions and generate more toxic hydroxyl radicals (·OH) and subsequently damage lipid contents in plasma membranes. Moreover, ferroptosis is thought to be more immunogenic than apoptosis. Ferroptotic cell death can potentiate immunogenic cell death (ICD), promote antitumor immunity, and thus enhance responses to immunotherapy. However, ferroptosis can be suppressed by antioxidant enzymes including GPX4 or system Xc-, which are responsible for GSH biogenesis. Thus, the development of ferroptotic nanotechnologies by either ion employment or disruption of antioxidant systems offers a potent strategy for cancer therapy [8–10].

Zoledronic acid (Zol), a bisphosphonate used to treat bone diseases such as osteoporosis, also exerts pleiotropic antitumor effects, including antiproliferative, antiangiogenic, antimetastatic, and immunomodulatory activities. Zol reverses the epithelial-mesenchymal transition (EMT) and growth of cancer cells through inactivation of nuclear factor (NF)-κB [11], and Zol inhibits osteoclastogenesis and bone resorption by suppressing receptor activator of NF-κB ligand (RANKL)‑mediated NF‑κB signaling. Moreover, Zol promotes M1 macrophage polarization, while it reverses M2-like tumor-associated macrophages (TAMs) and remodels the immunosuppressive TME [12]. Studies reported the enhanced therapeutic efficacy of Zol combined with immunotherapy in different malignancies [13, 14], shedding new light on improving cancer treatments. However, its therapeutic efficacy is limited by a short half-life and low plasma concentration, reducing its therapeutic efficiency. Furthermore, systemic injection of Zol causes severe side effects, which limits its clinical application. Nanoparticles offers a potential solution by enhancing Zol’s distribution and concentration in tumors, and prolonging its therapeutic effects. Fabricated Zol-based nanoplatforms were shown to possess promising efficacies in targeting bone metastasis, ameliorating cancer-associated osteolysis, and enhancing bone regeneration [15–17]. Moreover, Zol-loaded nanorods improved radiotherapy by synergistically inducing ICD [18].

**Fig. 1:**
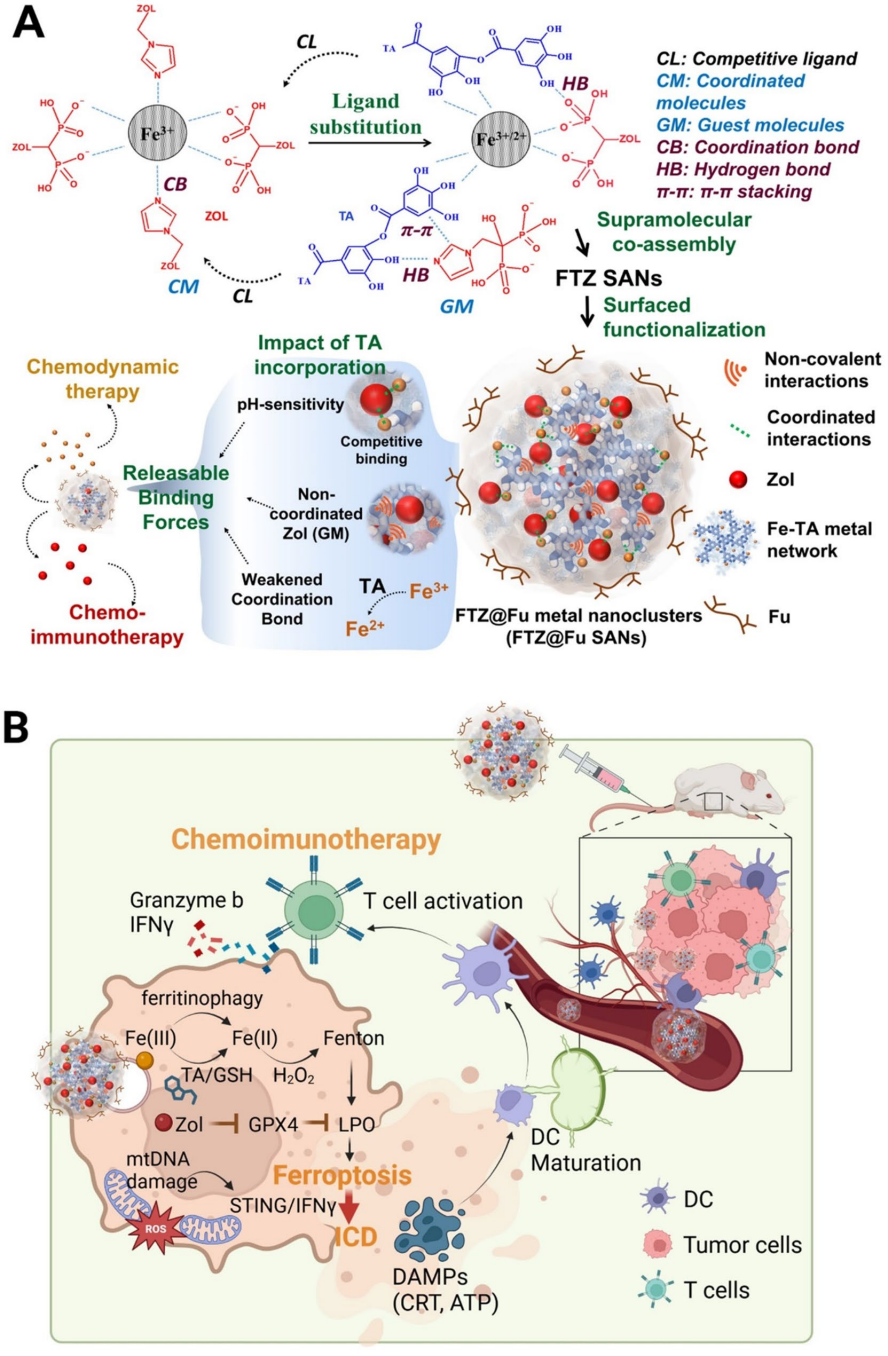
Illustration of the theranostic FTZ@Fu supramolecular-assembled nanoparticles (SANs) suppressing tumor metastasis and enhancing antitumor immunity. (**A**) The schematic diagrams illustrating the synthesis of FTZ@Fu SANs and their multifunctional antitumor mechanism to achieve efficient chemoimmunotherapy for cancer treatments. (**B**) The fucoidan coating of FTZ SANs (FTZ@Fu) exhibits an enhanced binding capability towards metastatic tumor cells. FTZ@Fu SANs are sensitive to the acid tumor microenvironment, and the release of tannic acid promotes Fe^3+^ reduction and triggers the Fenton reaction accompanied by inducing ferritinophagy. Subsequently, Zol promotes ferroptosis and induces mitochondrial DNA damage to further enhance immunogenic cell death. As a results, FTZ@Fu SANs attenuate tumor immunity and reinstate immune perception to suppress the tumor’s distant metastasis and therefore synergistically enhances tumor immunotherapy

The bisphosphonate, Zol, can form metal nanocomplexes (MNCs) with ferric chloride. However, the high stability of the formed complex of ferric chloride-Zol (FZ) MNCs hinders the release of Zol. In this study, we developed a novel supramolecular metal-organic ligand nanoplatform for sustained Zol delivery. Supramolecular self-assembly derived materials are constructed based on weak and reversible noncovalent interactions, such as hydrogen bonding, metal coordination, hydrophobic attractions, van der Waals forces, π − π stacking, and electrostatic interactions. The inherent reversibility of these interactions ensures that the assemblies can adapt and respond to specific stimuli, enhancing the targeted delivery and controlled release of therapeutic agents [19]. Thus, the competitive ligand tannic acid (TA) was used in this study to synthesize Fe-TA-Zol supramolecular assembled nanoparticles (FTZ SANs) through the coordination-driven self-assembly of Fe^3+^, TA, and Zol. TA is a polyphenol compound which can interacts with ferric chloride via metal-ligand coordination and disassociates in a pH response manner [20]. The interaction of TA and ferric chloride also benefits the release of Zol in an acidic TME and in lysosomes, and TA is also involved in iron redox cycling of Fenton-based oxidation which further catalyzes formation of the Fenton reaction and triggers ferritinophagy. Importantly, Zol not only suppresses tumor growth but also enhances LPO by promoting ferroptosis accompanied by induction of ICD. Finally, the pyrogallol group of TA also benefits the coating of fucoidan via hydrogen bonding in FTZ@Fu SANs, which further enhances binding specificity and internalization towards metastatic cancer cells (Fig. 1). Fucoidan was shown to inhibit tumor growth and cancer invasion and metastasis by targeting different oncogenic pathways [21, 22]. Our previous studies have demonstrated the binding of fucoidan onto p-selectin displays its potential for nanoparticle development [23–25]. This novel nanoplatform exhibited a synergistic inhibitory mechanism and multifunctional antitumor characteristics with enhanced chemodynamics, suppression of tumor metastasis, and potentiation of immune checkpoint blockage (ICB) to achieve efficient chemoimmunotherapy. The innovative approach provides proof of concept that smart, biocompatible fucoidan-based SANs can empower sweeping antitumor function and could serve as a reliable strategy for fighting cancer metastasis and recurrence clinically.

## Results and discussion

### Preparation and characterization of FTZ@Fu MNCs

Fig. 1A shows the schematic diagram for synthesis of the FTZ SAN as a drug-delivery nanoplatform. Zol as an organic ligand containing imidazole and bisphosphonate groups, which facilitates coordination with various metal ions to form metal nanocomplexes (MNCs). However, the strong binding affinity of Fe^3+^ towards Zol leads to the formation of highly stable Fe^3+^-Zol metal nanocomplexes (FZ MNCs). This high stability hinders the release of Zol from the MNCs, posing a challenge for biomedical applications that require controlled release of the compound. In the present work, FTZ SANs were first prepared to solve the problem of low anticancer activity of FZ MNCs due to its poor Zol release ability. Metal-phenolic networks (MPNs) are coordination complexes that form through interactions between metal ions (Cu^2+^, Zn^2+^, Fe^3+^, etc.) with phenolic ligands (quercetin, EGCG, TA, etc.) [20]. Inspired by the aforementioned metal-phenolic ligand coordination reaction, TA was used as a competitive ligand to replace Zol. This ligand substitution converted Zol from a coordination molecule into a guest molecule, thereby weakening the Fe-Zol binding strength (Fig. 1A). FTZ SAN was formed by the supramolecular assembly of Fe(III), TA, and Zol via coordination bonds (chelation) and non-covalent interactions, including hydrogen bonds, electrostatic interactions, and π–π stacking interactions. The coordination bonds and non-covalent interactions contributed to the dynamic nature of FTZ SANs, enabling the supramolecular system to be assembled and disassembled in response to external stimuli, such as pH changes. The stimulus-responsive behavior of FTZ SANs was particularly useful for targeted delivery and triggered release of therapeutics in the acidic environment of tumor tissues. Furthermore, the surface decoration of fucoidan (Fu) onto the FTZ SANs rendered the nanoparticles (FTZ@Fu) with an enhanced affinity towards tumor by virtue of specific interaction between fucoidan and P-selectin overexpressed on cancer cells. The fucoidan coated on FTZ@Fu SANs significantly enhances their affinity for metastatic tumor cells, thereby improving targeting specificity and reducing tumor aggressiveness.

Fig. 1B shows the multifunctional antitumor mechanism of FTZ SANs to achieve efficient chemoimmunotherapy for cancer treatments. Upon delivery of FTZ@Fu SANs into tumor sites, the low pH TME can induce the disassembly of Fe-TA-Zol coordination bonds. TA as a reductant promotes Fe^3+^/Fe^2+^ conversion in the acidic TME, which is beneficial for catalyzing H_2_O_2_ into highly toxic hydroxyl radicals to boost Fenton reaction cycle. The binding affinity of Fe^2+^ toward organic ligands like TA and Zol is notably weaker than that of Fe^3+^. Thus, the redox cycle introduces dynamic changes in the structural integrity of the TA-Fe-Zol coordinate metal-ligand networks through the TA-mediated Fe^3+^/Fe^2+^ redox cycle, thereby enabling a redox-responsive and tunable drug release. The hydroxyl radicals produced by the FTZ@Fu SANs could increase the level of ROS and decrease GSH content. The massive accumulated hydroxyl radicals is critical for the consumption of GSH to inhibit GPX4 and promote an overwhelming amount of LPO for enhanced ferroptosis. Concurrently, Zol enhances ferroptosis and stimulates ICD, further amplifying cell death and promoting a robust antitumor immune response.

**Fig. 2:**
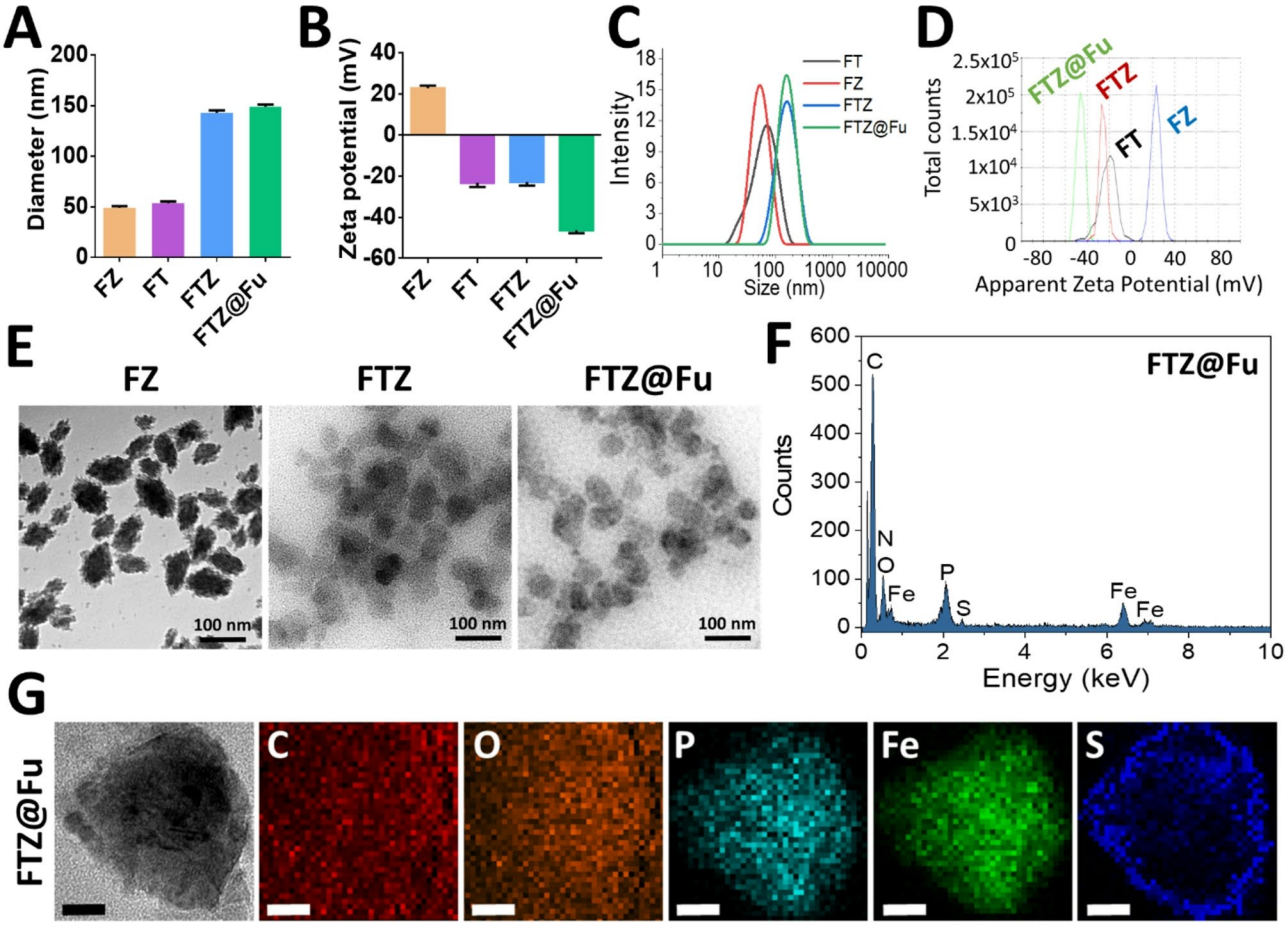
Establishment and characterization of supramolecular assembled nanoparticles. (**A**) Mean particle size, (**B**) ζ-potential, (**C**) Size distribution, and (**D**) ζ-potential distribution of different formulations (*n* = 3). (**E**) TEM images. (**F**) Elemental composition analysis, and (**G**) mapping images obtained by TEM energy-dispersive X-ray spectroscopy (EDS) after embedding in ultrathin epoxy resin (Scale bar = 20 nm)

Fe-TA MPN-based nanomaterials are of significant interest due to their potential applications in drug delivery. The structural properties, including the particle size, ζ-potential, and morphology, are critical in determining the stability, functionality, and performance of MPN nanomaterials in biomedical and drug-delivery applications. The NPs profiling and drug loading efficacies were shown in Fig. 2A and Table 1, average particle sizes of the Fe-TA metal-phenolic networks (FT MPNs) and Fe-Zol nanocomplexes (FZ MNCs) were 49 and 51 nm, respectively, as measured by DLS. The ζ-potential of FT MPNs was − 25 mV (Fig. 2B), indicating negatively charged surfaces due to the deprotonation of phenolic groups on TA. However, the ζ-potential of FZ MNCs became positive (21 mV), although the phosphate group on Zol was also deprotonated. Despite the deprotonation of phosphate groups on Zol contributing a negative charge to the surface of FZ MNCs, the predominance of Fe^3+^ allowed the MNCs to maintain a net positive ζ-potential. The predominance of Fe^3+^ has direct impact on further interaction of the Fe-Zol complex with other competitive ligands, such as TA. Accordingly, FZ MNCs could be further combined with TA to obtain FTZ SANs. The particle size of FTZ SANs increased to 145 nm, and the ζ-potential of NPs changed from positive potential (21 mV) to negative potential (-20 mV). The changes in ζ-potential was due to the binding of a large amount of the competitive ligand (TA). The substantial increase in FTZ SAN size suggests that ligand substitution through the incorporation of TA significantly alters the Fe-Zol coordination structure, resulting in a supramolecularly assembled Fe-TA-Zol network that is less compact than the networks found in FT MPNs and FZ MNCs. The hydrodynamic particle size of FTZ SANs after the fucoidan coating (FTZ@Fu SANs) increased slightly from 140 to 150 nm while the ζ-potential decreased from − 20 to -45 mV (Fig. 2A, B**)**. The slight increase in the hydrodynamic particle size after fucoidan coating indicates successful surface modification of FTZ SANs. This minor size increase suggests that the fucoidan formed a thin layer around the NPs without significantly altering their core structure. The substantial decrease in the ζ- potential confirms the presence of negatively charged fucoidan on the NP surface. The sulfate and carboxyl groups on fucoidan contributed to the more-negative charge, demonstrating its effective attachment. The size distribution and ζ-potential curves show that the particles of FT MPNs, FZ MNCs, FTZ, and FTZ@Fu SANs were well dispersed, and the surface charges were uniformly distributed (Fig. 2C, D). TEM images reveal that the FTZ SANs and FTZ@Fu SANs exhibit a lower density and a predominantly spherical or irregular morphology compared to the denser, more-compact FZ MNCs (Fig. 2E). This morphological shift is attributed to the introduction of TA, which likely disrupted the dense Fe-Zol coordination network through competitive binding with Fe³⁺, thereby inducing reassembly into a looser supramolecular structure. This structural loosening is supported by DLS and ζ-potential measurements, which revealed a disproportionate increase in the particle size and a reversal of the surface charge, reflecting a reduced packing density and altered surface chemistry. Furthermore, EDS was used to analyze the elemental composition and distribution within FTZ SANs and FTZ@Fu SANs. As shown in Fig. 2F, the EDS spectra show signals for Fe at 6.4 keV and P at 2.0 keV, confirming the combination of Fe and Zol. Signals for O and C appeared at 0.5 and 0.3 keV, respectively, corresponding to the TA and Zol components, indicating effective incorporation of Fe, TA, and Zol into the FTZ SAN NPs. Elemental mapping images show a uniform distribution of Fe, P, O, and C throughout the NPs (Fig. 2G**)**. The even distribution of signals corresponding to these elements indicates effective coordination between Fe^3+^, TA, and Zol. The EDS spectrum of FTZ@Fu SANs shows an additional S signal at 2.3 keV (Fig. 2F**)**. Elemental mapping images of FTZ@Fu SANs reveal a distinct S signal forming a circular pattern around the FTZ core (Fig. 2G**)**. This observation strongly supports the successful coating of fucoidan onto the surface of FTZ SANs. This modification not only enhanced the NPs’ stability in suspension but also imparted the bioactive properties of fucoidan, such as a tumor-targeting ability.

**Table 1:** Encapsulate efficiency and loading content

	EE (%)		LC (µg/mg NPs)	
	Zol	Fe	Zol	Fe
FTZ	91.3 ± 4.5	34.9 ± 3.2	496.8 ± 24.5	38.4 ± 3.5
FZ	99.8 ± 1.2	99.7 ± 0.7	814.2 ± 9.7	164.5 ± 1.2
FTZ@Fu	90.1 ± 6.1	46.1 ± 4.5	440.8 ± 29.8	45.6 ± 4.5

^1^ Value presented as mean ± standard division (*n* = 3)

^2^ EE, encapsulate efficiency; LC, loading content

**Fig. 3:**
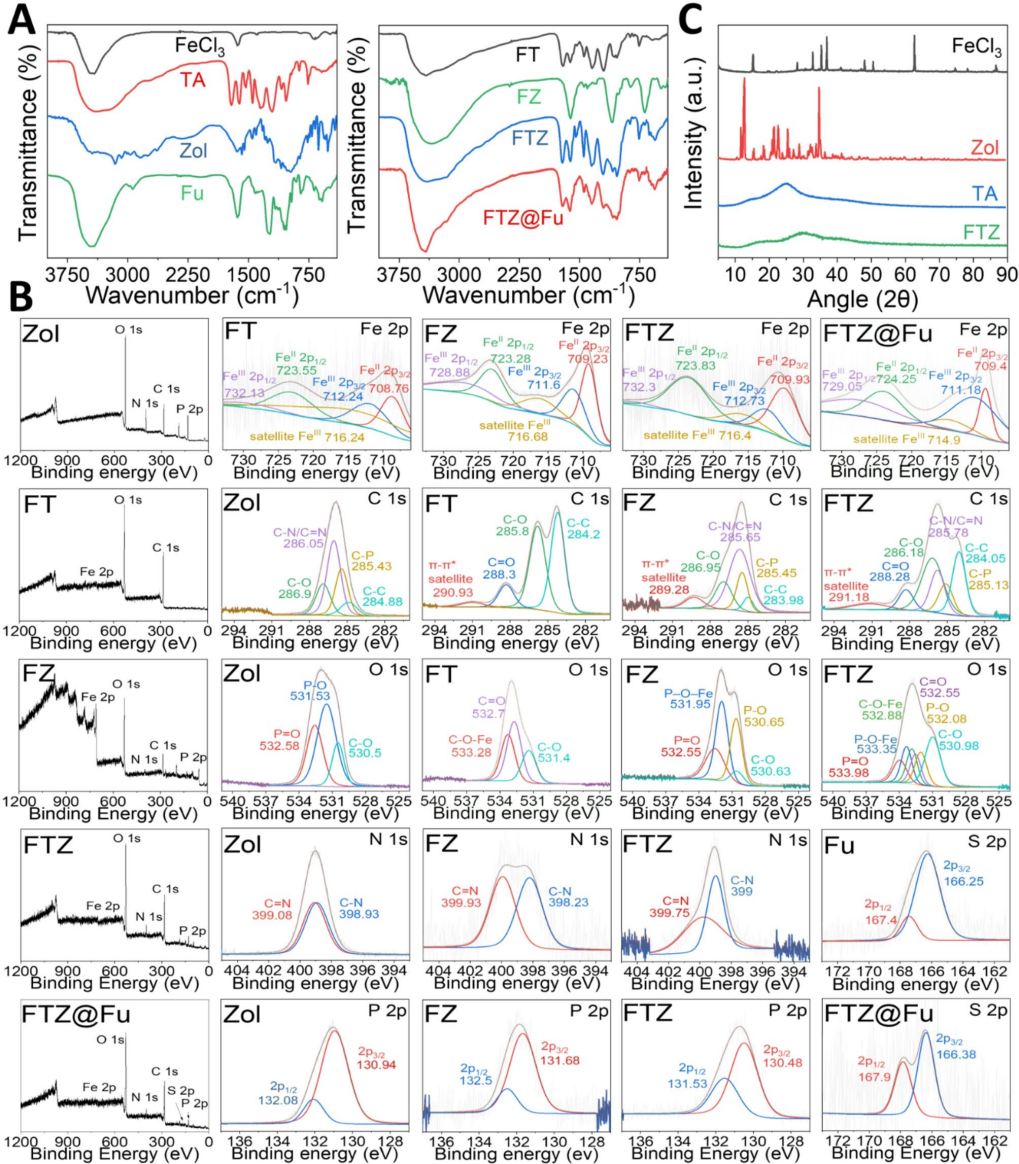
Characterization of supramolecular assembled nanoparticles. (**A**) FT-IR spectra, (**B**) XPS analysis, including survey spectrum and high-resolution spectra of Fe 2p, C 1s, O 1s, N 1s, P 2p, and S 2p, and (**C**) XRD patterns

FTIR spectroscopy provides valuable insights into the coordination between ferric ions and TA in the formation of MPNs. In the FTIR spectrum of pure TA (Fig. 3A), the broad band at around 3324 cm^− 1^ of O-H stretching vibrations represents hydrogen-bonded hydroxyl groups. Upon coordination with Fe^3+^, this band shifted to a higher wavenumber (3420 cm^− 1^) and decreased in intensity, reflecting that hydrogen bond had weakened due to the involvement of hydroxyl groups in the Fe-TA complexation. Aromatic C = C stretching vibration of TA (1619 and 1449 cm^− 1^) shifted slight after formation of Fe-TA coordination complexes (1612 and 1443 m^− 1^) due to enhanced π-π stacking interactions, suggesting closer packing of the aromatic rings. The appearance of two peaks at around 596 and 519 cm^− 1^ were associated with Fe-O stretching assigned to the reaction between galloyl groups of TA and Fe^3+^ [26], confirming the formation of the metal-phenol coordination network. These spectral changes provide direct evidence of structural modifications occurring in the FT MPNs, highlighting the key role of phenolic hydroxyl groups in the complexation process.

Zol is a bisphosphonate drug containing phosphate and imidazole groups, which can interact with metal ions to form coordination complexes. The phosphate groups in Zol showed two characteristic absorption bands assigned to P = O stretching vibrations at 1168 cm^− 1^ and P–O stretching vibrations at 1092 and 981 cm^− 1^ [27]. Upon coordination with ferric ions, these bands shifted to 920–1200 cm^− 1^ and broaden due to the formation of Fe-O-P bonds, indicating that the phosphate groups participated in metal coordination. The peaks at 1500–1700, and 1454 cm^− 1^ were assigned to the vibrations of imine/alkene (C = N/CH = CH) groups of the imidazole ring in Zol [27]. These bands shifted to 1615 and 1448 cm^− 1^ in FZ MNCs, suggesting coordination between imidazole nitrogen and Fe^3+^, and enhanced π-π stacking interactions between imidazole rings. The appearance of bands at around 484 cm^− 1^ corresponded to Fe–O stretching, providing direct evidence of ferric ion coordination. These spectral shifts and new bands confirmed the formation of Fe-Zol complexes through phosphate and imidazole interactions.

We further investigated interactions within the FTZ SANs for Fe-TA-Zol supramolecular assembly. This self-assembly involves coordination, hydrogen bonding, and π-π stacking between metal ions (Fe^3+^), a polyphenol (TA), and a bisphosphonate (Zol), where each component contributes unique functional groups to the structure. In the spectrum of FTZ SANs, the broad O-H and N-H stretching bands in the range of 3000–3600 cm^− 1^ reveals the formation hydrogen bonding due to supramolecular interactions between the phenolic groups of TA and the imidazole/phosphate groups of Zol. Furthermore, the characteristic P = O and P–O stretching vibrations of Zol appeared at 1209, 1086, and 1035 cm^− 1^, respectively. These band shifts suggested the formation of Fe-TA-Zol coordination instead of Fe-Zol coordination (1120 cm^− 1^) due to ligand substitution through the incorporation of the competitive ligand (TA) **(**Fig. 1A**)**. The coordination of Fe with both TA and Zol is further confirmed by shifting the characteristic Fe-O vibration band from 484 cm^− 1^ to 554 cm^− 1^ and increasing the absorption strength. Additionally, the peaks assigned to the vibrations of C = C/C-C and C = N/C-N bonds of TA and Zol shifted to 1613, 1546, 1447 and 1349 cm^− 1^, revealing the formation of TA-Zol π–π interactions and Fe-imidazole (Fe-N) chelation bonds in FTZ SANs. These spectral shifts and new bands confirmed the formation of Fe-TA-Zol coordination complex and highlight the role of phenolic, phosphate, and imidazole groups in metal-ligand coordination, hydrogen bonding, and π-π stacking **(**Fig. 1A**)**, which are responsible for supramolecular assembly of Fe^3+^, TA and Zol to form stable FTZ SANs. Furthermore, the formation of coordination bonds between Fe^3+^, TA and Zol enhances the proximity of aromatic rings, promoting π-π stacking interactions that contribute to the structural stability and functionality of the complex. In the FTIR spectrum of fucoidan (Fig. 3A), sulfate ester groups were identified by the characteristic absorption band around 1220–1260 cm⁻¹ corresponding to S = O stretching vibrations, and another characteristic absorption band at ca. 800–850 cm⁻¹ associated with C-O-S bending vibrations. However, in the FTZ@Fu SAN spectrum, characteristic absorption bands of fucoidan were not clearly visible due to the small amount and thin layer of fucoidan coating the NPs. 

Fig. 3B shows XPS spectra of FT MPNs, FZ MNCs and FTZ SANs, which offers valuable insights to investigate Fe-TA, Fe-Zol and Fe-TA-Zol coordination reaction and supramolecular interaction within these systems. The overall XPS spectra of FT MPNs, FZ MNCs and FTZ SANs has confirmed the presence of C, N, O, Fe, and P elements in these nanomaterials. The Fe 2p spectrum comprised two main peaks of Fe 2p_3/2_ and Fe 2p_1/2_ combined with vibrating satellite peaks. In the deconvoluted spectra of Fe 2p, the peaks at 710.7 and 723.4 eV were attributed to Fe^II^2p3/2 and Fe^II^2p1/2, while the peaks at 716.4 eV was attributed to Fe^III^2p satellite peaks. The peaks shifted in the Fe 2p spectra of FT MPNs, FZ MNCs and FTZ SANs, indicating that Fe^3+^ effectively binds to the ligands (TA and Zol) to form Fe-TA, Fe-Zol and Fe-TA-Zol coordination complexes. The deconvoluted C 1s spectrum of TA shows the deconvolved peaks at 284.6 eV (C–C/C = C), 286.3 eV (C–O), 288.8 eV (C = O) and 292.8 eV (π → π*), while the spectrum of Zol shows the deconvolved peaks at 284.9 eV (C-C), 285.4 eV (C-P), 286.1 eV (C-N/C = N), 286.9 eV (C–O), corresponding to the phenolic groups of TA and the carbon backbone of Zol. Upon complexation, the carbon atoms in the imidazole ring (C–N and C = N) or associated with the phosphonate groups (C–P) of Zol experience a shift in their binding energy. The peaks for C–N/C = N carbons and C–P carbon shifts to a lower binding energy, reflecting the electron donation from the nitrogen atoms to Fe and redistribution of electron density around the C-P bonds upon complexation of Fe^3+^ with Zol. The spectra of FT MPNs, FZ MNCs and FTZ SANs shows a new peak around 289 to 292 eV, suggesting that C atoms in TA or Zol can activate their π electrons into π* from the metal-ligand coordination, which contributes to the establishment of Fe-TA, Fe-Zol and Fe-TA-Zol networks. The deconvoluted N 1s spectrum of Zol shows the deconvolved peaks of C = N (399.1 eV), and C-N (398.9 eV), respectively. The spectra of FZ MNCs and FTZ SANs show the shifts in the C = N peaks, reflecting the coordination of this nitrogen atom with Fe to form Fe–N bonding structure. The P 2p spectrum of Zol shows deconvolved peaks at the binding energies of 130.9 and 132.1 eV, which correspond to the P 2p_3/2_ and P 2p_1/2_ signals. The shift of these peaks in the spectra of FZ MNCs and FTZ SANs indicates the phosphate groups of Zol coordinating with Fe^3+^ (132.5 eV, Fe–O–P bond). The deconvolved O 1s spectrum of FT MPNs shows the peaks at 533.2, 532.7 and 531.4 eV, corresponding to Fe–O–C, C = O and C–O bonds, indicating that Fe–TA coordination complexes were formed through the reaction between phenolic hydroxyl and Fe^3+^. The deconvolved O 1s spectrum of FZ MNCs shows the peaks at 532.6, 531.9, 530.7, and 530.6 eV, corresponding to P = O, Fe–O–P P–O, and C–O bonds, indicating that the oxygen atoms in the phosphonate groups participate in Fe coordination to form Fe–Zol complexes. The spectrum of FTZ SANs shows the shifts of Fe–O–C and Fe–O–P peaks, indicating that oxygen from both phosphate and hydroxyl groups are actively involved in the coordination of Fe^3+^ with TA and Zol. Overall, XPS analysis confirms the formation of a Fe-TA-Zol supramolecular assembly due to ligand substitution through the incorporation of the competitive ligand (TA) **(**Fig. 1A**).** supramolecular assembly through metal-ligand interactions between Fe^3+^, TA and Zol. The spectrum of FTZ SANs@Fu shows the deconvolved peaks of the S 2p signals at 167.9 and 166.4 eV corresponded to the binding energies of the S 2p_1/2_ and S 2p_3/2_ orbitals, which were attributed to sulfate ester groups on fu (Fig. 3B**)**, further confirm the successful coating of fu on the surface of FTZ SANs.

Fig. 3C shows XRD pattern of FeCl_3_, TA, Zol and FTZ SANs. The broad peak of low crystallinity observed in the spectrum of FTZ SANs around **12–47° (2θ)** represents the disordered packing of the Fe-TA-Zol coordination complex without extended crystallinity. This is because the ligand substitution on Fe-Zol coordination complex through coordination of the competitive ligand (TA) with ferric ions lead to form a metal-organic network with a high degree of disorder due to a rapid nucleation and growth process **(**Fig. 1A**)**. The lower crystallinity of FTZ SANs compare to FZ MNCs is characteristic of supramolecular assemblies that rely on dynamic and flexible metal-organic coordination bonds, as well as hydrogen bonding and π–π stacking, rather than rigid repeating crystal lattices. The highly amorphous structure constructed through ligand substitution and the change of coordinated Zol molecule to guest Zol molecule may enhance pH-dependent Zol and Fe release from FTZ SANs **(**Fig. 1A**)**.

**Fig. 4:**
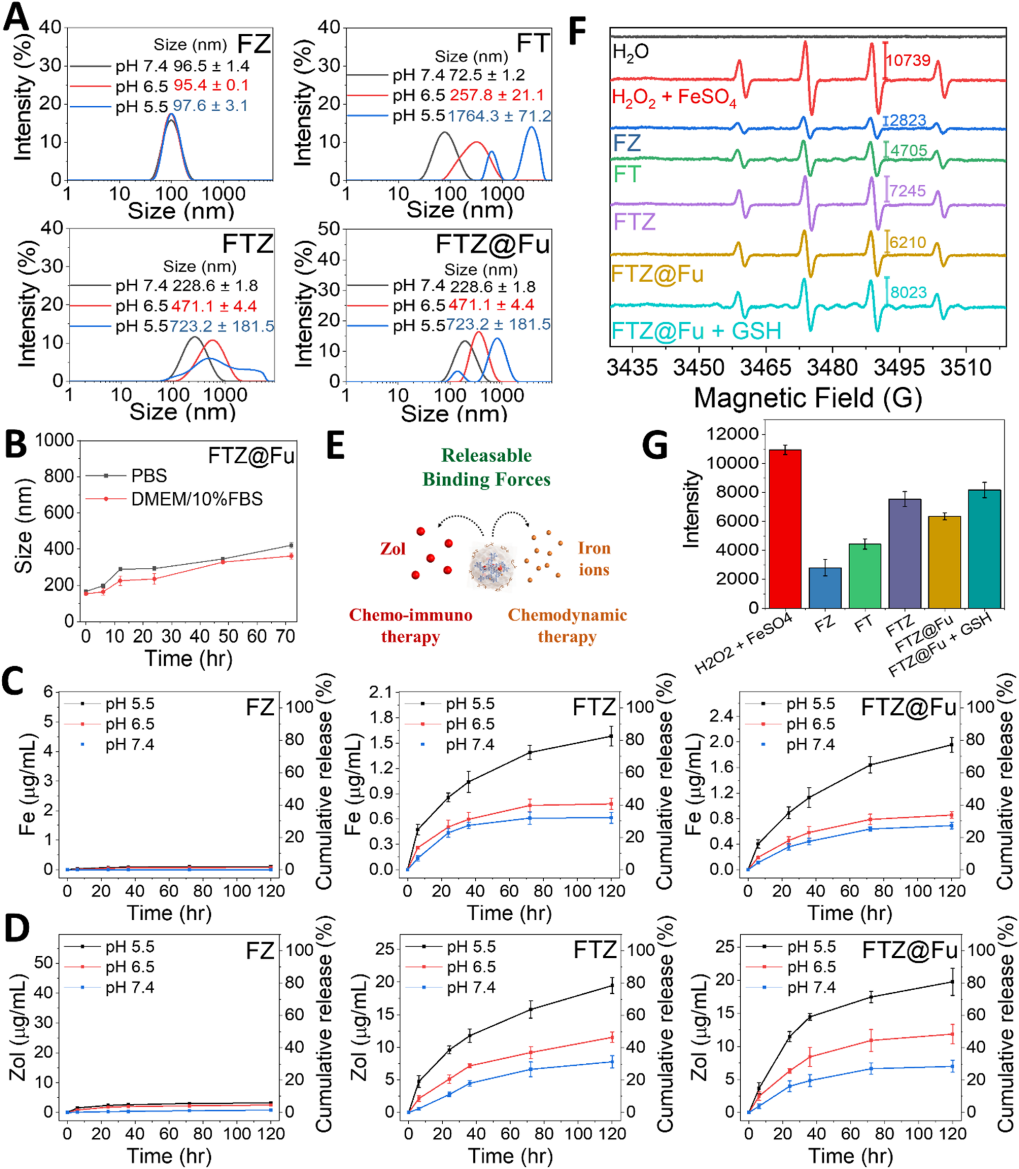
pH sensitivity and release profile. (**A**) Size distribution and (**B**) colloidal stability of FTZ@Fu under various physiologically relevant pH conditions at 37 °C (*n* = 4). (**C**) Cumulative Fe release and (**D**) cumulative Zol release under the same conditions. (**E**) Schematic illustration showing that both Fe and Zol could be released under mildly acidic conditions. (**F**, **G**) EPR analysis of hydroxyl radical (•OH) generation following a 72-h incubation of FTZ@Fu at pH 6.5 and subsequent reaction with H_2_O_2_

The pH-responsive behavior and drug-release characteristics of nanomedicines are crucial for their application in controlled drug delivery. FT MPNs exhibited significant pH sensitivity due to the nature of metal-phenol interactions within the network. At lower pH values, the environment is more acidic, leading to protonation of phenolic groups and weakening of metal-ligand bonds. Fig. 4A shows that the mean particle size of FT MPNs increased as the pH of the buffer medium decreased. This pH-dependent behavior can be attributed to the protonation state of TA and the coordination chemistry of Fe^3+^ under acidic conditions. Similarly, FTZ SANs and FTZ@Fu SANs demonstrated pH-responsive properties by increasing their mean particle sizes when the pH decreased, although the particle size of FZ MNCs was not affected by the environmental pH. These interesting results can be attributed to the high binding strength between Fe^3+^ and Zol. However, TA as a competitive ligand can compete with Zol to bind with Fe^3+^, causing destruction of the crystal lattice structure of FZ NCs and weakening of Fe-Zol coordination bonds through ligand substitution **(**Fig. 1A**)**, thereby creating a pH-responsive Fe-TA network in FTZ SANs. At lower pH values, TA is more protonated, reducing its ability to fully coordinate with Fe^3+^ ions. At a lower pH of 5.5, the increased proton availability weakens intramolecular interactions, leading to a more expanded FTZ SAN network that is less compact than at pH levels of 6.5 and 7.4. Consequently, the particle size and PDI of FTZ SAN are larger at pH 5.5 compared to those at pH 6.5 and 7.4 (Fig. 4A). In addition, the hydrodynamic diameter of FTZ@Fu SAN remained stable with less than 5% variation over 12 h in serum-containing culture medium (DMEM/10% FBS) (Fig. 4B). The results reveals the dual role of FTZ SAN in maintaining stability under physiological conditions and its targeted degradability in the TME, particularly under acidic pH and high GSH levels. The strong reducing nature of TA/GSH combination facilitates the conversion of Fe^3+^ to Fe^2+^, which is essential for inducing ferroptosis.

An increase in the size of NPs in acidic conditions could be advantageous for therapeutic applications, especially in cancer therapy, where the acidic TME could trigger size expansion and promote localized drug release. As shown in Fig. 4C and D, the release profiles of Fe³⁺and Zol from FZ MNCs remained relatively stable across various pH conditions (5.5, 6.5, and 7.4). This indicates that the coordination between Fe³⁺ and Zol in FZ MNCs is highly stable and unaffected by pH changes, ensuring consistent slow release behavior in both physiological and slightly acidic environments. However, the competitive ligand TA can compete with Zol to create a Fe-TA-Zol supramolecular self-assembled structures **(**Fig. 1A**)**. The release of Fe^3+^ ions and Zol from FTZ SANs was influenced by the pH of the surrounding environment. In acidic conditions, protonation of phenolic groups reduced the binding affinity of Fe^3+^ ions, leading to a higher release rate of iron. Therefore, under acidic conditions, such as those found in the TME, the FTZ and FTZ@Fu SANs exhibited more-pronounced responses. Hence, Fe^3+^ release was facilitated by the decreased binding affinity of Fe^3+^ to deprotonated phenolic groups of TA and phosphate groups of Zol. The release of Zol from FTZ and FTZ@Fu SANs was similarly enhanced in acidic conditions, driven by the weakened coordination between TA/Zol and Fe^3+^. This pH-sensitive release of both Fe^3+^ and Zol ensures that the therapeutic agents are preferentially delivered in acidic environments, such as tumor tissues, where their therapeutic action is most effective (Fig. 4E).

Fig. 4F and G shows EPR spectral analysis of the hydroxyl radicals generated by the Fenton reaction in the H_2_O_2_ + FTZ and H_2_O_2_ + FTZ@Fu SANs systems. In both systems, the strong signals corresponding to the •OH-DMPO adducts were observed at a pattern of 1:2:2:1 splitting. The ligand substitution in the Fe-Zol coordination complex, driven by the competitive coordination of TA with ferric ions, leads to the formation of an Fe-TA-Zol network, accompanied by partial reduction of Fe³⁺ to Fe²⁺ due to the intrinsic reducing activity of TA **(**Fig. 1A**)**. This redox activity enhances Fenton reactivity, as evidenced by an approximately 2.5-fold increase in hydroxyl radical (•OH) generation by FTZ compared to FZ (Fig. 4G), supporting the role of TA-mediated Fe redox cycling in sustaining the production of •OH radicals. The results from EPR spectral analysis provides evidence on hydroxyl radical formation and the catalytic performance of FTZ and FTZ@Fu SANs in the Fenton reaction. The results emphasizes the role of Fe²⁺ in catalyzing the production of hydroxyl radicals through Fenton and Fenton-like reactions, particularly in the presence of H₂O₂. In the TME, the reduction of Fe³⁺ to Fe²⁺ by TA and GSH drives this process, generating reactive hydroxyl radicals that cause oxidative stress. The EPR experiments demonstrate that Fe-TA systems, especially with GSH, significantly enhance the production of these radicals at mildly acidic conditions (pH 6.5), typical of the TME. The high levels of hydroxyl radicals lead to LPO, which sensitizes cancer cells to ferroptosis, a form of cell death driven by oxidative damage. This mechanism highlights the therapeutic potential of Fe-TA systems in promoting ferroptosis.

### FTZ@Fu MNCs targeted tumor cells and suppressed tumor growth

**Fig. 5:**
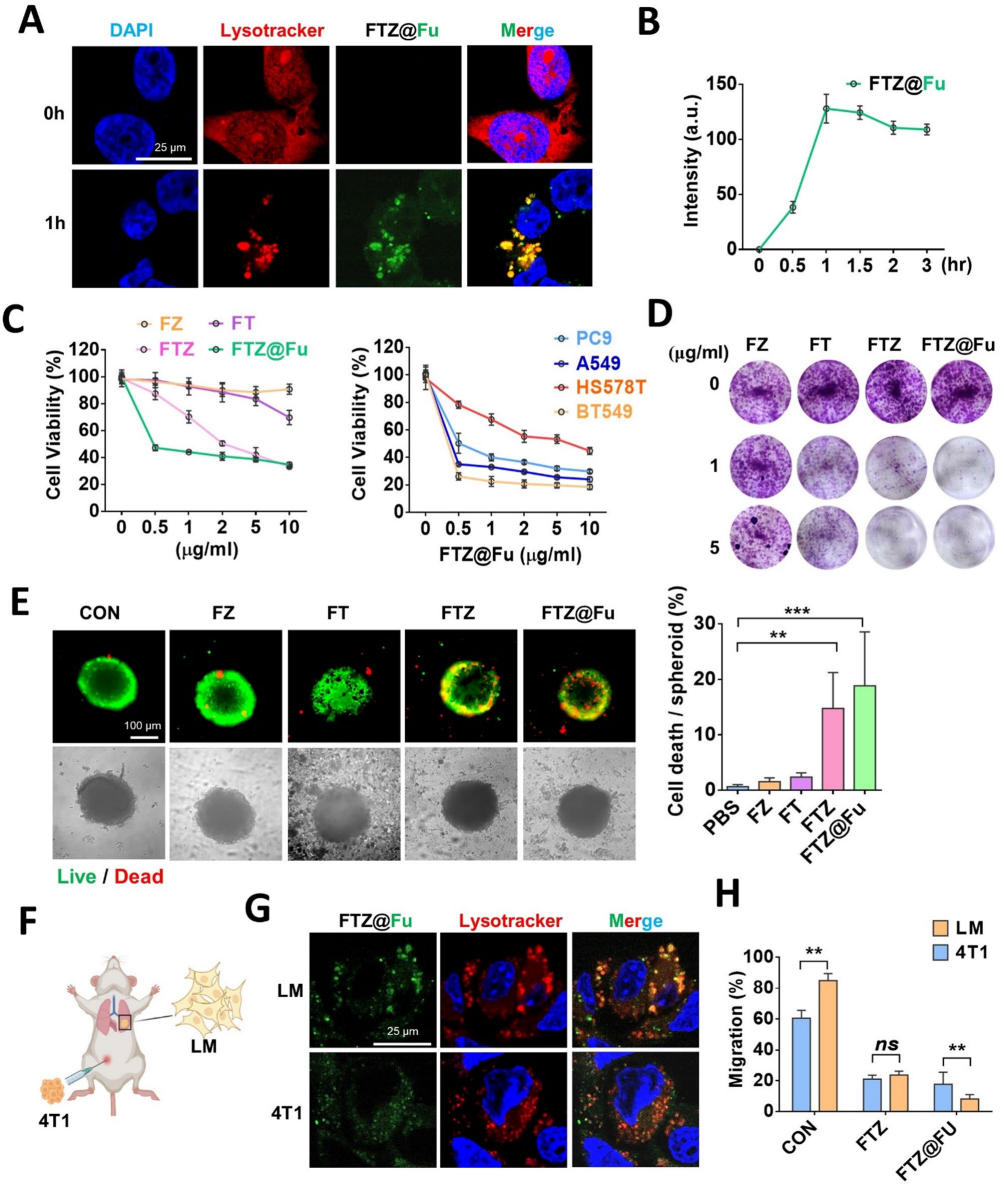
In vitro inhibitory effect of FTZ@Fu SANs on tumor cells. (**A**) CLSM images of cellular uptake of FTZ@Fu SANs in MDA-MB-231 cells. (**B**) Quantification of intracellular uptake in MDA-MB-231 cells treated with FTZ@Fu (5 µg/ml). (**C**) Growth inhibition of breast cancer (BT549 and Hs578T) and lung (A549 and PC9) tumor cells treated with various FTZ@Fu MNCs formulations for 48 h. (**D**) Colony-formation assay of various FTZ SANs in MDA-MB-231 cells. (**E**) Calcein-AM/EthD-III staining of live and dead 4T1 tumoroids after treatment with FZ, FT, FTZ, and FTZ@Fu. (**F**) Schematic diagram of the establishment of lung metastatic tumor cells from an orthotopic mammary tumor model. (**G**) CLSM images of cellular uptake of FTZ@Fu SANs by parental 4T1 and metastatic 4T1-LM cells. (**H**) Inhibitory effect of FTZ@Fu SANs on cell migration in parental 4T1 and metastatic 4T1-LM cells. Data are presented as the mean ± SD. * *p* < 0.05; ** *p* < 0.01; *** *p* < 0.001, assessed using an unpaired *t*-test

Cellular internalization of FTZ@Fu SANs by tumor cells was examined using CLSM. FTZ SANs were coated with fluorescein-conjugated fucoidan and treated cells for 0–3 h. As shown in Fig. 5A, fluorescent signals of FTZ@Fu SANs accumulated in lysosomes after 1 h of incubation in breast cancer MDA-MB-231 cells (Fig. 5A). Cellular uptake of FTZ@Fu was detected 0.5 h after treatment, increased to its maximum at 1 h, and persisted until 3 h (Fig. 5B). Moreover, results of the MTT assay showed that the viability of breast tumor cells was reduced by an increase of the fucoidan concentration in FTZ SANs, compared to the non-coated group (Supplemental Fig. S1A), indicating that the fucoidan coating increased tumor binding and promoted tumoricidal activity. We further compared the inhibitory effect of different formations on tumor growth, and we found that FZ and FT MNCs had no cytotoxic activity against MDA-MB-231 cells, while FTZ and FTZ@Fu substantially suppressed tumor growth (Fig. 5C). Moreover, FTZ@Fu also effectively inhibited tumor growth in lung adenocarcinoma (A549 and PC9) and breast cancer (Hs578T and BT549) cells (Fig. 5C), suggesting its antitumor potential was not dependent on a specific cell type. Similarly, the tumor colony-forming capability was significantly suppressed by FTZ and FTZ@Fu, compared to FZ and FT MNCs (Fig. 5D).

To further validate the therapeutic efficacy of FTZ@Fu, we treated 4T1 tumoroids with various NPs formulations and stained them with Live/Dead dyes. Consistently, FTZ and FTZ@Fu substantially promoted cell death in 4T1 tumoroids, while FZ and FT nanoclusters did not (Fig. 5E). Given that fucoidan was reported to suppress tumor invasion and metastasis [21], we isolated lung metastatic breast cancer cells using an orthotopic mammary tumor model and examined the effect of FTZ@Fu on metastatic tumor cells (Fig. 5F). Notably, FTZ@Fu increased binding activity towards metastatic 4T1-LM cells, compared to parental 4T1 cells (Fig. 5G). Moreover, the wound-healing migration assay showed that 4T1-LM cells had an enhanced migratory ability compared to parental cells. However, FTZ and FTZ@Fu effectively suppressed tumor migration of both parental and 4T1-LM cells (Supplemental Fig. S1B). Importantly, FTZ@Fu obviously enhanced an inhibitory effect on the invasiveness of 4T1-LM cells, compared to 4T1 cells, while FTZ SANs without fucoidan decoration exhibited no difference between 4T1 and 4T1-LM cells (Fig. 5H). These data highlight that fucoidan-coated FTZ SANs enabled a targeting capability towards metastatic tumor cells.

### FTZ@Fu SANs induce mitochondrial ROS production and promote ferroptosis

**Fig. 6:**
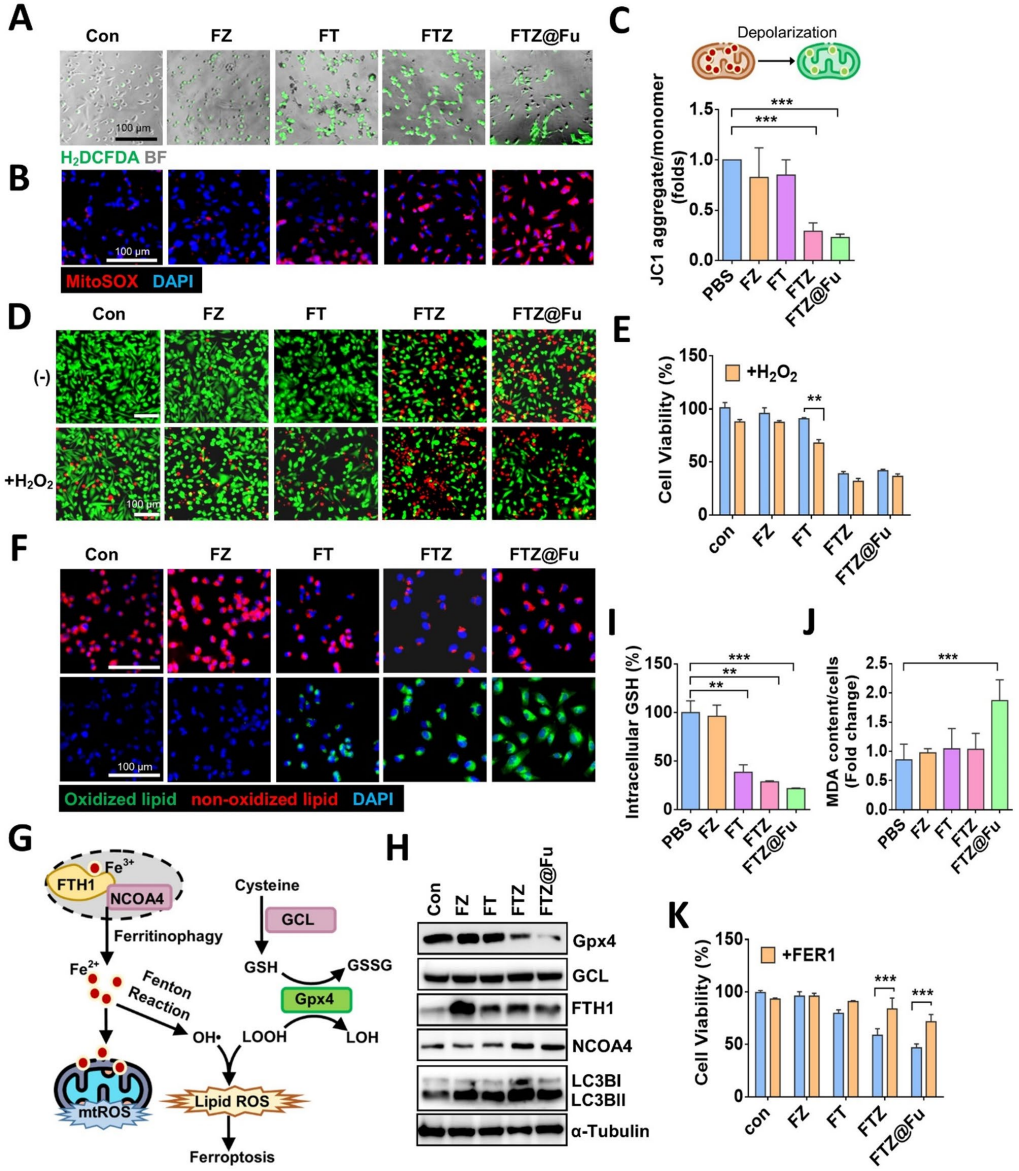
FTZ@Fu promotes ROS production and induces ferroptosis. (**A**) Fluorescent images of intracellular ROS production in MDA-MB-231 cells treated with FZ, FT, FTZ, and FTZ@Fu MNCs (5 µg/ml). BF, bright field. Bar, 100 μm. (**B**) Fluorescent images of mitochondrion-specific ROS production and (**C**) relative mitochondrial membrane potential in MDA-MB-231 cells treated with various MNCs (5 µg/ml) for 24 h. (**D**) Live/Death staining and (**E**) cellular viability of MDA-MB-231 cells treated with various MNCs in the presence of H_2_O_2_. (**F**) Fluorescent images of LPO in MDA-MB-231 cells treated with various MNCs. Red indicates non-oxidized lipids; green indicates oxidized lipids. (**G**) Intracellular signaling mechanism of ferritinophagy. (**H**) Western blot analysis of ferritinophagy-related protein level. (**I**) Relative fold of intracellular GSH content and (**J**) lipid peroxidation for malondialdehyde (MDA) assay in response to various MNCs. (**K**) Cellular viability of MDA-MB-231 cells treated with various MNCs in the presence or absence of the ferroptosis inhibitor, ferrostatin-1 (FER-1; 5 µM). Data are presented as the mean ± SD. * *p* < 0.05; ** *p* < 0.01; *** *p* < 0.001, assessed using an unpaired *t*-test

TA promotes the reduction of Fe^3+^ to Fe^2+^, which can further catalyze the Fenton reaction in the presence of hydrogen peroxide. To confirm the induction of ROS by the addition of TA in FTZ@Fu SANs, we stained MDA-MB-231 cells with DCFH-DA dye, a ROS-sensing probe (Fig. 6A). Fluorescent microscopy revealed that intracellular ROS increased in FT MPNs-treated cells, and similar results were obtained in FTZ- and FTZ@Fu SANs-treated cells (Fig. 6A). On the contrary, FZ MNPs showed no effect on ROS production (Fig. 6A). As mitochondrial redox imbalance is involved in Fenton reaction-mediated cell death, we stained cells with a mitochondrion-specific ROS probe and found that mitochondrial superoxide increased with FT treatment, and was obviously elevated in FTZ- and FTZ@Fu SANs-treated groups (Fig. 6B). We further examined the mitochondrial membrane potential (MMP) using JC-1 dye, and we validated that the MMP was significantly suppressed by FTZ and FTZ@Fu, but not by FZ or FT MNCs (Fig. 6C). These findings were similar to the aforementioned results that FZ and FT MNCs showed less cytotoxic effects. These data indicate that the reduction of Fe^2+^ by FT MNCs was not sufficient to elicit cell death; thus, cells were supplemented with different MNCs in the presence of hydrogen peroxide. Results showed that the addition of hydrogen peroxide significantly reduced cell viability and promoted cell death in FT MPNs-treated cells, compared to the FT alone-treated group (Fig. 6D, E). However, this inhibitory result was not competitive with FTZ and FTZ@Fu SANs, highlighting the anticancer potentiality of Zol beyond the Fenton reaction in this SAN nanoplatform.

Recent studies showed that Zol can promote cell death by triggering ferroptosis [13, 28, 29], which is caused by excess ferric ion accumulation and LPO. We thus examined the effect of FTZ SANs on ferroptotic cell death by staining with BODIPY C11 581/591, an LPO sensor to monitor ferroptosis. Similar to mitochondrial ROS levels, we observed that treatment with FZ MNCs had no effect on LPO, while treatment with FT MPNs slightly induced LPO. Importantly, FTZ and FTZ@Fu drastically promoted LPO (Fig. 6F). Fe^3+^ triggers ferritinophagy by cooperation with ferritin and nuclear receptor coactivator 4 (NCOA4), a cargo receptor that mediates degradation of ferritin in autophagosomes and contributes to iron accumulation. We thus further analyzed ferroptosis marker expressions and found that all of the nanoclusters increased the FTH1 protein level, while only FTZ and FTZ@Fu SANs upregulated NCOA4 expression (Fig. 6G). On the other hand, treatment with FTZ and FTZ@Fu obviously decreased GPX4 protein expression (Fig. 6H). Consistent results were obtained by immunofluorescent staining (Supplemental Fig. S2A). Because GPX4 acts as a mainstay in regulating ferroptosis by using GSH to eliminate peroxidized lipids, we found that intracellular GSH was significantly reduced by the addition of FT MPNs, but was enhanced by FTZ and FTZ@Fu SANs (Fig. 6I). Accordingly, we also analyzed the glutamate-cysteine ligase (GCL) catalytic (GCLC) subunit, a main subunit of GCL in GSH biosynthesis, while no obvious change in the GCLC protein level was found in the presence of FTZ@Fu treatment (Fig. 6G, H). Moreover, MDA assay indicated lipid peroxidation elicited by FTZ@Fu (Fig. 6J), and the reduced cell viabilities by FTZ@Fu were reverted in the presence of a ferroptosis inhibitor (Fig. 6K). Together, these data indicate that the release of ferric ions and TA by FT nanoclusters triggered ferritinophagy accompanied by induction of the Fenton reaction, while suppression of GPX4 by FTZ and FTZ@Fu SANs potentiated LPO and thus aggravated cell death.

### FTZ@Fu SANs induce antitumor immune responses

**Fig. 7:**
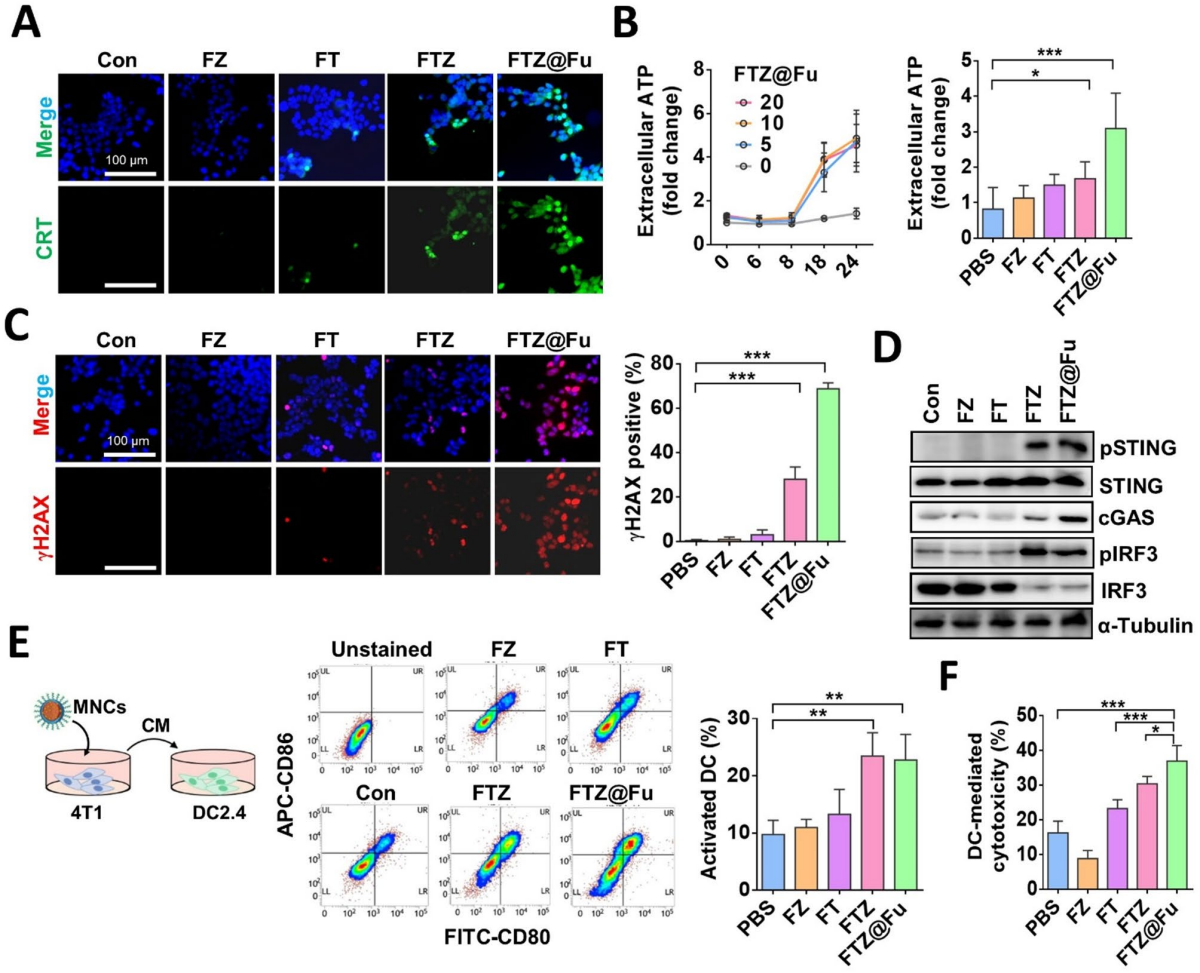
FTZ@Fu SANs induce immunogenic cell death and potentiate immune checkpoint blockage therapy. (**A**) CLSM images of CRT expression in MDA-MB-231 cells after being treated with FZ, FT, FTZ, and FTZ@Fu MNCs (5 µg/ml) for 24 h. Bar = 100 μm. (**B**) Extracellular ATP release by MDA-MB-231 cells treated with FTZ@Fu (0–20 µg/ml) (left panel) or different MNC formulations (right panel). (**C**) CLSM images of γH2AX expression in MDA-MB-231 cells after being treated with MNCs (5 µg/ml) for 24 h. Bar =100 μm. Quantification of γH2AX-positive cells is shown in the right panel. (**D**) Western blot showing activation of the sGAS/STING pathway in MDA-MB-231 cells treated with MNCs. (**E**) Flow cytometric analysis of dendritic cell maturation (left panel), and quantitative data are shown in the right panel. (**F**) Quantification of dendritic cell-mediated tumor cell phagocytosis by a flow cytometric analysis

Immunogenic cell death (ICD) is a form of cell death that can engage immunity, which leads to more-effective responses by eliciting antitumor immunity. ICD causes the release or surface exposure of a series of damage-associated molecular patterns (DAMPs). Recent studies reported that ferroptotic cells undergo ICD at an early stage during cell death [30–32]. We next examined ICD markers including Calreticulin (CRT) exposure (eat me signal) and extracellular ATP release (find me signal). Tumor cells were incubated with various MNCs, and results showed that FTZ and FTZ@Fu drastically induced CRT exposure in MDA-MB-231 cells (Fig. 7A). Moreover, FTZ@Fu increased extracellular ATP contents in a concentration-dependent manner, compared to FTZ and FT nanoclusters (Fig. 7B). Similarly, FTZ and FTZ@Fu treatments obviously induced expression of the γH2AX DNA damage marker (Fig. 7C). As our data showed that FTZ@Fu triggered mitochondrial oxidative stress, we further examined the stimulator of interferon genes (STING) pathway, which is reported to be triggered by mitochondrial DNA damage to mediate antitumor immunity and inflammatory responses [33, 34]. Notably, FTZ and FTZ@Fu treatments effectively stimulated phosphorylation of STING and IRF3, while cGAS was engaged by FTZ@Fu (Fig. 7D), demonstrating that FTZ-induced ferroptosis was capable of triggering SITNG activation which was augmented by Fucoidan. To validate whether FTZ MNCs-mediated ICD can activate dendritic cells (DCs), we treated 4T1 cells with various nanoclusters followed by coculture with DCs and then stained them with CD80/CD86, which are mature DCs markers. Results of flow cytometric analyses demonstrated that FTZ and FTZ@Fu SAN treatments promoted DCs maturation, compared to the FZ- or FT-treated groups (Fig. 7E). Moreover, the phagocytosis activity of DCs was significantly induced by FTZ and FTZ@Fu SANs (Fig. 7F). These data demonstrate the successful induction of ICD in tumor cells and subsequent promotion of DCs maturation and activation by FTZ@Fu. We further analyzed the effect of FTZ@Fu on TAMs, which are known to induce an immunosuppressive TME and promote tumor progression. Mouse RAW264.7 macrophages were pretreated with FTZ@Fu followed by incubation with 4T1 tumor conditioned medium (CM) (Supplemental Fig. S3A). Results showed that expression of the tumor CM-induced M2 macrophage marker, Arg1, was suppressed by FTZ@Fu. On the contrary, the M1 macrophage marker, iNOS, was increased by FTZ@Fu (Supplemental Fig. S3B), indicating the potential of FTZ@Fu in shaping polarization of TAMs toward an antitumor M1-like phenotype.

### FTZ@Fu SANs suppressed tumor metastasis and ameliorated the immunosuppressive TME

**Fig. 8:**
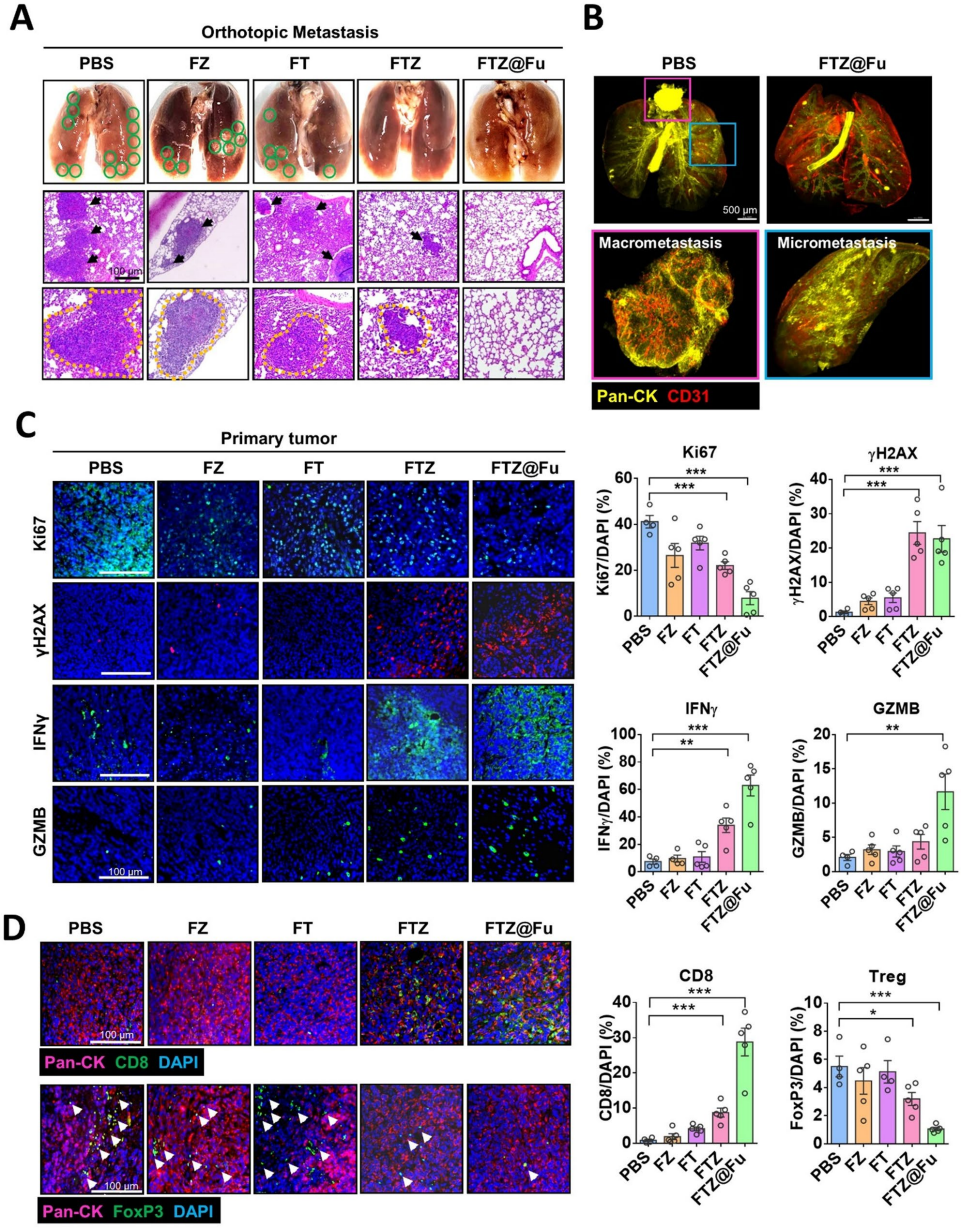
FTZ@Fu inhibits tumor metastasis and attenuates the immunosuppressive TME. (**A**) Hematoxylin and eosin (H&E) staining of spontaneous lung metastasis from orthotopically implanted 4T1-LM cells with different MNCs formulations treatments. Bar = 100 μm. Quantification of metastatic tumor colonies is shown. (**B**) 3D images showing tumor (PanCK) and vasculature (CD31) in mice lung. Enlarged pictures indicate micro- (blue area) and macrometastasis (pink area). (**C**) Representative images of the expressions of Ki67, γH2AX, IFN-γ, and granzyme b in nuclei (DAPI) in tumor tissues. Quantitative data are shown in the right panel. Bar = 100 μm. (**D**) Representative images of Treg and CD8 T-cell infiltration in tumor tissues by MNC treatment. Quantitative data are shown in the right panel. Bar = 100 μm. * *p* < 0.05; ** *p* < 0.01; and *** *p* < 0.001

To verify the therapeutic specificity of FTZ@Fu SANs against tumor metastasis. 4T1-LM cells were orthotopically injected into the mammary fat pad of BALB/c mice, and different formulations of FTZ SANs were applied. The ex-vivo data confirmed the tumor accumulation of FTZ@Fu after 24 h administration (Supplemental Fig. S4A), and serum biochemistry analyses of creatinine, bilirubin, albumin, and ALT showing no significant cytotoxicity after FTZ@Fu treatment for 3 weeks (Supplemental Fig. S4B). Moreover, no obvious systemic toxicity was observed in the kidneys, livers, and spleen (Supplemental Fig. S4C). After treatments, mice were sacrificed, and a histological examination of mice lung sections showed that FTZ@Fu exhibited the most effective therapeutic efficacy on tumor metastasis, compared to the FTZ-treated group (Fig. 8A). On the contrary, FZ and FT MNCs showed no inhibitory effect on tumor metastasis (Fig. 8A). Moreover, 3D digital-spatial images showed obvious macrometastatic tumor nodules enriched with tumor-associated vasculature observed in untreated mice lungs, compared to FTZ@Fu treatment (Fig. 8B). Additionally, IF staining indicated that FTZ@Fu treatment significantly reduced Ki67 expression, while it increased the γH2AX level, indicating that FTZ@Fu SANs suppressed tumor proliferation and induced DNA damage in tumor cells (Fig. 8C). Moreover, FTZ@Fu treatment significantly increased IFN-γ and granzyme b expressions (Fig. 8C), demonstrating the induction of antitumor immunity in metastatic tumor tissues. Consistently, the expression of tumor-infiltrating lymphocytes such as regulatory T cells (FoxP3^+^) was reduced, while CD8 + T-cell infiltration was increased by FTZ@Fu, compared to other MNCs formulations (Fig. 8D). Together, our data demonstrated that FTZ@Fu reshaped the immunosuppressive TME in metastatic tumor tissues.

### FTZ@Fu SANs reprogram antitumor immunity and potentiate immune checkpoint blockage therapy

**Fig. 9:**
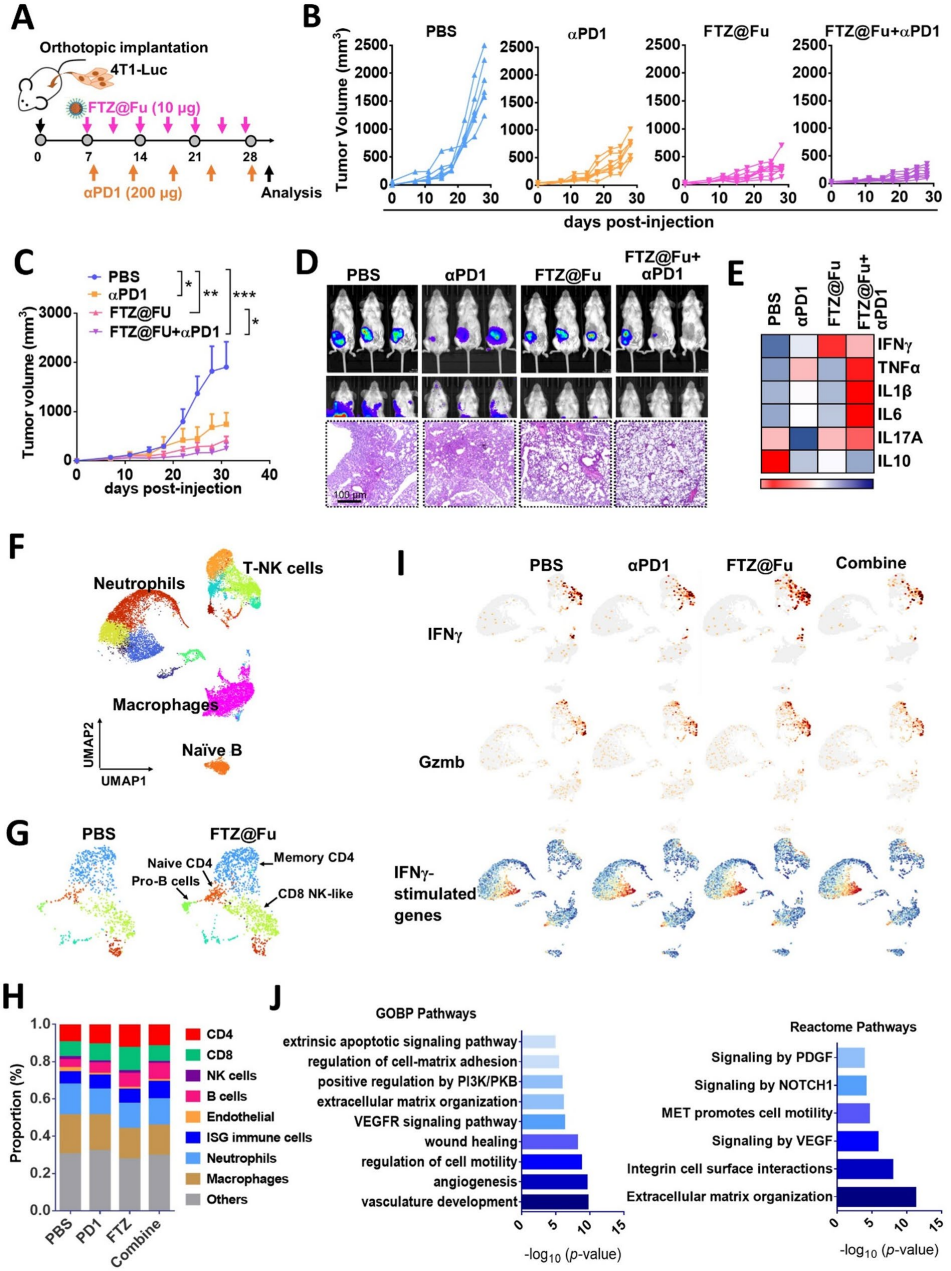
FTZ@Fu SANs reshape antitumor immunity and potentiate immune checkpoint therapy. (**A**) Schematic diagram of experimental and treatment schedules of Balb/c mice. (**B**) Growth curve showing individual tumor volumes over time of 4T1-Luc cells in mice (*n* = 7) treated with FTZ@Fu, anti-PD1, or their combination. (**C**) Mean ± SEM of tumor growth curve in each group. Significance was calculated using a one-way ANOVA and Holm-Sidak multiple-comparison tests; * *p* < 0.05; ** *p* < 0.01; and *** *p* < 0.001. (**D**) Representative images of IVIS luminescence signals in tumor-bearing mice treated with FTZ@Fu, anti-PD1, or their combination on day 25 (upper panel), and representative images of pulmonary histology by H&E staining (lower panel). Bar = 100 μm. (**E**) Heatmap plot of intratumoral inflammatory cytokine levels by a Multi-Plex Immunoassay. (**F**) Unsupervised clustering of different transcriptome dataset using Seurat. Data are shown by UMAP projection. (**G**) FTZ@Fu treatment increased T-NK cell components. (**H**) Proportion of cell identified in response to different MNCs formulation. (**I**) Expression levels of IFNγ, Granzym B, and ISG signature in different MNCs-treated group. (**J**) Gene Ontology and Reactome analyses of enriched pathways in FTZ@Fu group compared to PBS

To further determine whether FTZ@Fu SANs-elicited antitumor immune responses showed therapeutic promise with immunotherapy combinations, 4T1-Luc cells were orthotopically injected into the mammary gland of Balb/c mice, and tumor-bearing mice were administrated FTZ@Fu (10 µg; two times/week) combined with immune checkpoint blockage by an anti-PD1 antibody (100 µg; every 5 days) (Fig. 9A). Results revealed that tumor-bearing mice treated with anti-PD1 alone had a 55% reduction in tumor growth, while FTZ@Fu treatment induced 70% tumor remission. Importantly, combined treatment achieved a remarkably vigorous tumor inhibition effect of up to 85% (Fig. 9B-C). No significant change in weight loss was seen after these treatments (Supplemental Fig. S5A). Consistently, images of an in vivo imaging system (IVIS) showed that combined treatment with FTZ@Fu and an anti-PD1 antibody obviously reduced bioluminescence in the tumor region, compared to monotherapy (Fig. 9D). Moreover, results of IVIS images and lung histology showed that both FTZ@Fu and FTZ@Fu/anti-PD1 combined treatments effectively suppressed tumor dissemination in the lungs (Fig. 9D), suggesting that FTZ@Fu has the ability to prime a strong antitumor immune response. Indeed, tumor extracts were subjected to a Multi-Plex Immunoassay, and results revealed that FTZ@Fu treatment increased secretion of the immune modulatory cytokine, interferon (IFN)-γ, and FTZ@Fu combined with anti-PD1 treatments promoted proinflammatory cytokines, including interleukin (IL)-1β, IL-6, and tumor necrosis factor (TNF)-α production. Conversely, the anti-inflammatory cytokine, IL-10, decreased with combined therapy (Fig. 9E), highlighting that FTZ@Fu enhanced the antitumor immune response and improved the therapeutic efficacy when combined with ICB. Moreover, results of the IHC assay showed that GPX4 expression levels in primary tumor tissues were robustly inhibited by FTZ@Fu, but not by anti-PD1 (Supplemental Fig. S2B-C). Conversely, FTZ@Fu induced 4-hydroxynonenal (4-HNE) production, an LPO marker, demonstrating the induction of ferroptosis in tumor tissues (Supplemental Fig. S2C). IHC staining of GPX4 also illustrated the elevation of GPX4 in lung metastatic tumor tissues, and more interestingly at the primary invasive front but not the primary interior site (Supplemental Fig. S2B). Our data were in line with a recent study that the GPX4 inhibitor reprogrammed the TME and conferred synergistic effects in combination with ICB [35]. Accordingly, IHC data showed that combination of FTZ@Fu and anti-PD1 treatments enhanced the expressions of CD11c (the dendritic cell marker), CD86 (the M1 macrophage marker), and IFNγ expression (Supplemental Fig. S5B). Collectively, these data provide compelling evidence that the induction of ferroptosis by FTZ@Fu SANs offers a promising opportunity for improving immunotherapy.

We further conducted single-cell (sc)RNA-Seq to gain insights into the antitumor immune response by FTZ@Fu SANs. After treatment, tumor tissues were dissected and disassociated, excluding dead cells, followed by 10× genome analysis (Fig. 9F), and UMAP plots showed individual cell clusters (Fig. 9F-G). Notably, we found that the proportions of T-natural killer (NK) cell subgroups, including naïve and memory CD4, CD8, and CD8-NK-like T cells, increased after FTZ@Fu treatment, compared to the vehicle group (Fig. 9G-H). On the contrary, the proportion of endothelial cells decreased (Fig. 9G-H). We further found that expressions of IFN-γ and granzyme b by CD8 NK-like clusters increased in FTZ@Fu and combined treatment groups, compared to the PBS or anti-PD1 groups (Fig. 9I). Moreover, the IFN-γ-stimulated gene (ISG) signature, which is involved in antigen presentation, antiproliferative activities, and stimulation of adaptive immunity, was concomitantly elevated (Fig. 9I). Our data also showed that FTZ@Fu SANs treatment had a potent effect of increasing CD4/CD8 T-cell proportions, while the combination of FTZ@Fu and ICB therapy greatly increased B-cell and ISG populations, suggesting that stimulating innate immunity by FTZ@Fu SANs subsequently promoted a specific immune response, thus potentiating ICB therapy. Interestingly, we analyzed pathways significantly enriched by FTZ@Fu treatment, and Gene Ontology results showed that vasculature development, angiogenesis, wound healing, and extracellular matrix organization signatures were affected (Fig. 9J). Similarly, the Reactome pathway analysis showed that the VEGF, MET, NOTCH, and PDGF signaling pathways, which are associated with tumor vascularization and tumor mobility, were disrupted after FTZ@Fu treatment (Fig. 9J). These data echo our immunohistochemistry examination in which FTZ@Fu treatment impacted tumor angiogenesis, demonstrating that FTZ@Fu SANs affected tumor angiogenesis and reshaped antitumor immunity to enhance ICB therapy.

FTZ SANs utilize TA as a competitive ligand to drive supramolecular self-assembly while enabling the pH-responsive release of Fe^3+^, TA, and Zol. FTZ@Fu SANs offer significant advantages over conventional ferroptosis-inducing nanoplatforms by integrating a multifunctional antitumor mechanism that synergistically enhances both ferroptosis and immunotherapy. Unlike traditional ferroptosis agents [36, 37], which primarily focus on iron delivery or ROS generation, FTZ@Fu SANs employ a pH-responsive Fe-TA-Zol coordination system that disassembles in the acidic tumor microenvironment (TME). This triggers the controlled release of Fe^3+^, facilitating a TA-mediated Fe^3+^/Fe^2+^ redox cycle that continuously catalyzes H_2_O_2_ into highly toxic hydroxyl radicals, promoting efficient GSH depletion and GPX4 inhibition while reinforcing the Fenton reaction cycle. Moreover, the redox-responsive nature of the TA-Fe-Zol network enables the controlled release of Zol, further enhancing therapeutic efficacy. Unlike many ferroptosis-inducing nanoplatforms that primarily rely on ROS accumulation, FTZ@Fu SANs uniquely integrate ferroptosis with immunogenic cell death (ICD) induction via Zol, amplifying tumor cell death and stimulating a robust antitumor immune response. This dual therapeutic strategy positions FTZ@Fu SANs as a highly effective ferroptosis-inducing nanoplatform with superior efficacy, specificity, and immunomodulatory potential compared to conventional approaches.

Ferroptosis has gained much attention in recent tumor therapy studies due to its induction of tumor immunogenicity and high selectivity toward tumor growth, and it thus reserves therapeutic vulnerability to overcome resistance to traditional therapy and offers a potent strategy for enhanced cancer immunotherapy. Some tumors, especially triple-negative breast cancer cells, are reported to resist ferroptosis by increasing GPX4 [38]. GPX4 overexpression is closely related to a poor prognosis of TNBC [39], and GPX4 inhibition boosts ferroptosis and triggers an antitumor immune response [35, 40]. GPX4 is a selenocysteine-containing protein with strong antioxidant activity, that plays a critical role in anti-ferroptosis. Several studies reported that GPX4 expression confers drug resistance to tumor cells, while its inhibition increased vulnerability in persistent cells and prevented the acquisition of drug resistance. Notably, the epithelial-mesenchymal transition (EMT) inducer, Zeb1, was identified as a regulator of GPX4 expression in therapy-resistant cells [41]. Additionally, GPX4 expression was found to be correlated with antioxidant proteins in castration-resistant prostate cancer patients [42, 43], and its overexpression was linked to metastasis and several cancer types. To address cancer resilience, various strategies have been developed to target GPX4, including the use of natural products, medicinal compounds, repurposed drugs, and nanoparticles [44, 45]. In our study, we observed that GPX4 expression was upregulated in metastatic tumor tissues. Furthermore, we demonstrated that encapsulation of Zol in FTZ@Fu MNCs effectively suppressed GPX4, which resulted in increased lipid peroxidation and aggravation of ferroptosis. Our study showed that GPX4 was elevated at the invasive front of the primary tumor site and overexpressed in metastatic tumor tissues. We also demonstrated that FTZ@Fu SANs effectively induced ferroptosis by inducing ferritinophagy and suppressing GPX4. Moreover, FTZ@Fu SANs induced ICD to further activate the STING/IFN pathway and restore tumor immunogenicity. The polysaccharide, fucoidan, was reported to inhibit tumor aggressiveness and angiogenesis, and exert immunomodulatory effects. We previously developed a fu-based photodynamic nanosystem to suppress programmed death ligand 1 (PD-L1) expression and tumor immunity for treating TNBC [25]. In our study, the fucoidan-decorated FTZ@Fu SANs enhanced binding activity towards metastatic breast cancer cells and effectively suppressed tumor aggressiveness. Moreover, in vivo studies demonstrated that FTZ@Fu treatment significantly inhibited breast tumor growth and dissemination and suppressed metastatic tumor growth. The combination of FTZ@Fu with checkpoint blockage enhanced the therapeutic efficacy and reshaped the immunosuppressive TME with negligible systemic toxicity. Zol is well-characterized to reduce osteolysis induced by bone metastasis, and Zol-loaded nanosystems enabled targeted delivery to TAMs. It should be noted that bone metastases commonly occur in patients with breast, lung, or prostate cancer. Our FTZ SANs effectively induced cytotoxicity in breast and lung cancer cells, and it showed benefits by inducing ferroptosis and enhancing ICD. These data indicate that fucoidan and Zol can synergistically inhibit tumor aggressiveness, induce ferroptosis, and modulate tumor immunity, opening a promising avenue for overcoming tumor metastasis. Therefore, it would be worth further investigating the therapeutic potential of FTZ@Fu SANs against bone metastasis.

In summary, we developed a novel approach by supramolecular co-assembly of TA, ferric chloride, Zol, and decoration with fucoidan to form FTZ@Fu SANs. We demonstrated that FTZ SANs effectively suppressed tumor growth by inducing chemodynamics and ferroptosis, which is capable of triggering ICD. The coating of fucoidan on FTZ SANs enhanced the binding affinity to metastatic tumor cells and augmented the antitumor immune response. Thus, this nanoplatform exhibited versatile therapeutic advantages including (1) enhancing binding activity towards metastatic tumor cells, (2) synergetic chemodynamic/ferroptosis/ICD, and (3) reshaping tumor immunity. This SANs platform not only supplies a paradigm for overcoming tumor metastasis but also elicits antitumor immunity for an effective chemoimmunotherapeutic combination, paving the way for successful cancer treatment.

## Materials and methods

### Materials

Tannic acid and Iron(III), Zol, and chloride hexahydrate (FeCl_3_) were purchased from Sigma-Aldrich (USA). Fucoidan (from Laminaria japonica, 40 kDa molecular weight, 22% L-fucose, 28% sulfate content, 8% carboxyl groups) was purchased from NOVA Pharma & Liposome Biotech.

### Preparation and characterization of FTZ@Fu SANs

FT: 1 mL of 5 mM FeCl_3_ solution was mixed with 1 mL of 5 mM tannic acid solution under vigorous stirring for 5 min. The resulting nanoparticles were collected by centrifugation at 6000 rpm and resuspended in deionized (DI) water (molar ratio of Fe^3+^: TA = 1:1). FZ: 1 mL of 50 mM FeCl_3_ solution was combined with 1 mL of 5 mM Zol solution under vigorous stirring for 10 min, followed by heating in a water bath at 70 °C for 90 min. The nanoparticles were collected by centrifugation at 12,000 rpm and resuspended in DI water (molar ratio of Fe^3+^: Zol = 10:1). FTZ: 3 mL of 5 mM tannic acid solution was premixed with 1 mL of 5 mM Zol solution for 3 min, after which 1 mL of 50 mM FeCl_3_ solution was added under vigorous stirring for 5 min. The nanoparticles were harvested by centrifugation at 6000 rpm and resuspended in DI water (molar ratio of Fe^3+^: TA: Zol ≈ 10:3:1; mass ratio ≈ 6:18:1). FTZ@Fu: FTZ nanoparticles were diluted to a concentration of 1 mg/mL, then mixed with a 1% fucoidan solution at a weight ratio of 10:1, followed by vigorous stirring for 5 min. Excess Fu was removed by centrifugation at 6000 rpm (mass ratio of FTZ: Fu = 10:1).

### Characterization

Hydrodynamic diameter and ζ-potential were analyzed using a Zetasizer Ultra (Malvern, Worcestershire, UK). The morphology of the particles was observed via transmission electron microscopy (TEM) using a H-7700 TEM system, following dispersion and drying on copper grids under vacuum. For detailed structural analysis, TEM samples were prepared through resin embedding, sectioning, and examined using an FEI Tecnai™ G2 F-20 S-TWIN microscope operated at 200 kV (FEI COMPANY, USA). Fourier transform infrared (FT-IR) spectra were obtained using the potassium bromide pellet method, and measurements were conducted with an FTS-155 FT-IR spectrometer. The crystalline structure of the nanocomplexes was characterized using a D2 Phaser X-ray diffractometer (Bruker, Germany), equipped with a nickel filter and CuKα radiation (λ = 0.154 nm) at 15 kV and 20 mA. Scans were performed in continuous mode with a scan rate of 5.3°/min over a 2θ range from 5° to 80°. X-ray photoelectron spectroscopy (XPS) analysis was carried out using a high-resolution XPS system (ULVAC-PHI, PHI Quantera II, Japan). For electron spin resonance (ESR) analysis of hydroxyl radicals (•OH), samples were mixed with H_2_O_2_ (1 mM), FeSO_4_ (2 mM), and 5,5-dimethyl-1-pyrroline N-oxide (DMPO, 100 mM), then immediately transferred into capillaries. ESR measurements were conducted using an EMX 10/12 spectrometer (Bruker) under the following conditions: resonance frequency of 9.42 GHz, microwave power of 6.33 mW, modulation frequency of 100 kHz, and modulation amplitude of 1.0 G.

### Releasing profile of Fe and Zol

Samples (1 mg/mL) were placed in 3 kDa cutoff dialysis bags and immersed in saline buffer at specific pH values, with the buffer volume set to 19 times the sample volume. The concentrations of Fe and Zol were quantified by determining the amounts of Fe and phosphorus (P) after sample digestion in concentrated nitric acid using a high-pressure digestion vessel at 180 °C for 60 min. The digested solution was then diluted and analyzed by inductively coupled plasma mass spectrometry (ICP-MS/MS, Agilent 8900, Agilent, Waldbronn, Germany) integrated with an Agilent 1260 Infinity Quaternary LC System (Agilent, Waldbronn, Germany). The encapsulation efficiency (%) and the encapsulated amount were measured using the same method. Unencapsulated materials were separated using 3 kDa cutoff ultrafiltration tubes (Vivaspin 2^®^, Sartorius, USA).

### Cellular uptake assay

Human breast cancer MDA-MB-231 and mouse mammary tumor 4T1 cells were cultured in Dulbecco’s modified Eagle medium (DMEM) supplemented with 10% fetal bovine serum (FBS; Corning) and 1% penicillin/streptomycin with 5% CO_2_ at 37 °C. 4T1-LM cells were isolated from tumor lung metastasis by an orthotopic injection of 4T1 cells in the mammary fat pad of Balb/c mice in our previous study [2]. Cells were seeded on a chamber slide at a density of 5 × 10^4^ cells/well and treated with 1 µM FITC-conjugated fucoidan-decorated FTZ@Fu MNCs for 0 ~ 60 min. Cells were stained with lysotracker dye (Invitrogen), and nuclei were counterstained with DAPI. FTZ@Fu was visualized under a laser confocal scan microscope (Leica).

### Cell viability and colony-formation assays

Human breast cancer MDA-MB-231, BT549, MCF7 cells, and lung cancer A549 and PC9 cells were seeded at 3 × 10^4^ cells/well in a 48-well plate and treated with different concentrations of MNCs for 48 h. Cell viability was assessed by adding 3-(4,5-dimethylthiazol-2-yl)-2,5-diphenyltetrazolium bromide (MTT; 10 µg/ml) for 4 h. The formazan crystals were dissolved in isopropanol, and the absorbance was measured at an optical density (OD) of 570 nm on a Spark M10 plate reader (Tecan Group). Cell viability was calculated and is presented as a percentage relative to untreated cells. A colony-formation assay was performed by seeding 10^5^ cells/ml in six-well plates and treating them with MNCs for 10 days.

### Wound-healing scratch assay

4T1 and LM cells were obtained from our previous study. Briefly, cells were seeded at density of 10^6^ cells/well into six-well plates, and a wound was scratched using a 200-µl tip. Cells were treated with MNCs for 18 h, and visualized under an inverted microscope. The wound gap area was quantified using Image J software, and cell migration was defined as the gap area after treatment relative to 0 h.

### 3D tumorsphere formation and live/dead assay

Mouse 4T1 cells (5 × 10^3^) were seeded in a 96-well plate precoated with 1% Pluronic-F127 (Sigma-Aldrich), centrifuged at 1000 rpm for 5 min, and incubated at 37 °C for 96 h to form tumoroids. Then, tumoroids were treated with MNCs for another 96 h, stained with a Calcein AM/EthD-III staining kit (Biotium) according to the manufacturer’s instructions, and observed under a fluorescence microscope.

### Animal model

All animal models were carried out based on institutional guidelines by the Animal Care and Use Committee of Taipei Medical University (LAC2023-0058). Murine breast cancer 4T1-Luc cells (2 × 10^6^) were mixed with 50% Matrigel and inoculated on the fourth mammary fat pad of female 8-week-old BALB/c mice (*n* = 6) (National Laboratory Animal Center, Taiwan) for 1 week. Mice were randomly divided into individual groups and intravenously injected with phosphate-buffered saline (PBS), FTZ@Fu (500 µg/kg) twice a week, an anti-mouse programmed death 1 (PD1) antibody (Clone 29 F.1A12™, 10 mg/kg body weight (BW); Bio X Cell) every 5 days, or combined treatment for a total of 4 weeks. The tumor size was calculated based on the equation: (width) × (length)^2^ / 2. Live images of mouse tumor and lung metastasis were captured using an in vivo imaging system (IVIS) bioluminescence system. Additionally, 10^5^ murine breast cancer 4T1-LM cells were inoculated into the left mammary fat pad of female 8-week-old BALB/c mice (*n* = 5) (National Laboratory Animal Center) for 1 week. Mice were randomly divided into individual groups and intravenously injected with PBS, FZ, FT, FTZ, and FTZ@Fu (500 µg/kg BW) twice a week for a total of 4 weeks. On day 30 after tumor inoculation, mice were sacrificed, and the lungs, spleen, liver, kidneys, and tumor were harvested for histological examination.

### Whole mouse lung clearing and immunolabeling

Mice were transcardially perfused with ice-cold phosphate-buffered saline (PBS), followed by a perfusion with a solution of 4% paraformaldehyde and epoxy resin. The lung was then dissected and immersed in the same PFA/epoxy perfusion solution, incubating at 4 °C for 48 h. Subsequently, the tissue was transferred to an epoxy cross-linking activation solution at 37 °C for 24 h to complete fixation. The clearing process was carried out over a period of 6 days at 45 °C until the tissue became translucent. Each lung tissue sample was labeled with 40 µL of mouse anti-PanCK monoclonal antibody (Cell Signaling, #4545), followed by 40 µL of goat anti-CD31 polyclonal antibody (R&D Systems, AF3628). Subsequently, fluorescently conjugated secondary antibodies were applied in a 1:2 primary to secondary molar ratio (Jackson ImmunoResearch).

### Biodistribution and systemic toxicity analyses

Rhodamine-conjugated fucoidan was coated with FTZ SANs and intravenously injected into the tail vein of tumor-bearing mice for 6 h. Mice were sacrificed and organs were harvested, and the accumulation of FTZ@Fu was detected using an IVIS bioluminescence system. For systemic toxicity examinations, tumor-bearing mice were administered FTZ@Fu (500 µg/kg BW) for 4 weeks. On day 30, mice serum was harvested and AST, ALT, BUN, and creatinine levels, and H&E sections of typical major organs were monitored to evaluate the systemic toxicity at the end of treatment.

### Ferroptosis detection

Cells (10^5^/ml) were seeded on cover slides and treated with different formulations of FTZ MNCs for 24 h. Lipid ROS were measured by staining with BODIPY 581/591-C11 (Invitrogen), a lipid peroxidation sensor probe, for 30 min. Peroxidized lipids were observed by excitation and emission at 488/510 nm under a fluorescent microscope (Zeiss). To detect lipid peroxidation, a malondialdehyde (MDA) assay was applied. In brief, cells were treated with different MNC formulations for 24 h. Cells were lysed and subjected to an MDA colorimetric assay kit (Elabscience) according to the manufacturer’s instructions.

### Intracellular and mitochondrial ROS detection

Cells (10^5^/ml) were seeded on cover slides and treated with different formulations of FTZ MNCs for 24 h. Intracellular ROS were detected by incubating with a 2’-7’dichlorofluorescin diacetate (DCFH-DA) probe (Sigma) for 30 min and monitored using a fluorescent microscope (Zeiss). To measure mitochondrial superoxide, live cells were treated with FTZ MNCs for 24 h and incubated with a MitoSOX red probe (Invitrogen) as per the manufacture’s instructions.

### Immunofluorescent staining

Cells (10^5^/ml) were seeded on cover slides and treated with different formulations of FTZ MNCs for 24 h. For the CRT exposure measurement, cells were fixed with 4% paraformaldehyde, blocked with 3% BSA/TBST, stained with FITC-conjugated CRT (ab196158, Abcam) overnight at 4 °C, and observed using a confocal laser scanning microscope (CLSM; Leica). For γH2AX, cells were incubated with a primary antibody against γH2AX (GTX127340, GeneTex) overnight at 4 °C and then incubated with an anti-rabbit CF488 secondary antibody (406404, BioLegend) for another 1 h.

### Flow cytometric analysis for dendritic cell (DC) maturation

4T1 tumor cells (10^5^/ml) were seeded into six-well plates and treated with MNCs for 24 h. Cells were washed with fresh medium and cocultured with mouse DC2.4 DCs (10^5^/ml) for an additional 24 h. Cells were trypsinized, suspended in FACS buffer (PBS/1% FBS), and stained with FITC-CD80 and APC-CD86 antibodies (Biolegend) for 1 h at 4 °C followed by analysis with flow cytometry (BD Biosciences).

### Extracellular ATP measurement

In a 48-well plate, 3 × 10^4^ cells/well were treated with different concentrations of MNCs for 24 h. After treatment, culture supernatants were collected and analyzed using a RealTime-Glo™ Extracellular ATP Assay (Promega) as per the manufacture’s instructions; luminescence was detected using a multimode spectrum microplate reader.

### Western blot analysis

Detailed methodology is shown in our previous work [1]. Specific primary antibodies are as following: GCLC (ab53179, Abcam), GPX4 (GTX54095, GeneTex), NCOA4 (#66849, Cell Signaling Technology), LC3B (#83506, Cell Signaling Technology), FTH1 (#4393, Cell Signaling Technology), and a STING pathway sampler kit (#38866, Cell Signaling Technology).

### Immunohistochemistry (IHC)

Detailed methodology is shown in our previous work [25]. Specific primary antibodies are as following: Ki67 (GTX16667, GeneTex), γH2AX (GTX127340, GeneTex), IFN-γ (ab216642, Abcam), granzyme b (ab4059, Abcam), Pan-keratin (#4545, Cell Signaling Technology), CD8 (GTX16696, GeneTex), and FoxP3 (#12653, Cell Signaling Technology).

### Single cell RNA sequencing analyses

4T1 tumor tissue isolated from female Balb/c mice were digested by using gentleMACS™ Dissociator (Miltenyi Biotec) following dead cell and debris removal kits (Miltenyi Biotec) to obtain single cell suspension according to the manufacture’s instructions. Single cell sequencing preparation using Cell Ranger on a 10x Genomics Chromium Controller following manufacturers protocol. scRNA-Seq libraries were sequenced using Illumina (San Diego, CA, USA) NextSeq 500. Integrated analysis were performed by using Seurat.

### Statistical analysis

Each experiment was performed in triplicate, and results are expressed as the mean ± standard deviation (SD) unless otherwise specifically indicated. The significance of the difference from the respective controls for each experimental test condition was assayed using an unpaired *t*-test. * *p* < 0.05, ** *p* < 0.01, and *** *p* < 0.001 were regarded as significant differences compared to the indicated group. Statistical analyses were carried out using GraphPad Prism software.

## Electronic supplementary material

Below is the link to the electronic supplementary material.

Supplementary Material 1

## Data Availability

No datasets were generated or analysed during the current study.
