# Supplementary material for: Fucoidan-decorated metal-zoledronic acid nanocomplexes suppress tumor metastasis by inducing ferroptotic cell death and enhancing cancer immunotherapy
Source: J Nanobiotechnology. 2025 Jun 2;23:405. doi: 10.1186/s12951-025-03473-0 (PMC12128240; doi:10.1186/s12951-025-03473-0)
Supplement: Supplementary file 1 — Supplementary Material 1 [file 12951_2025_3473_MOESM1_ESM.docx]

**Supplementary Information**

**
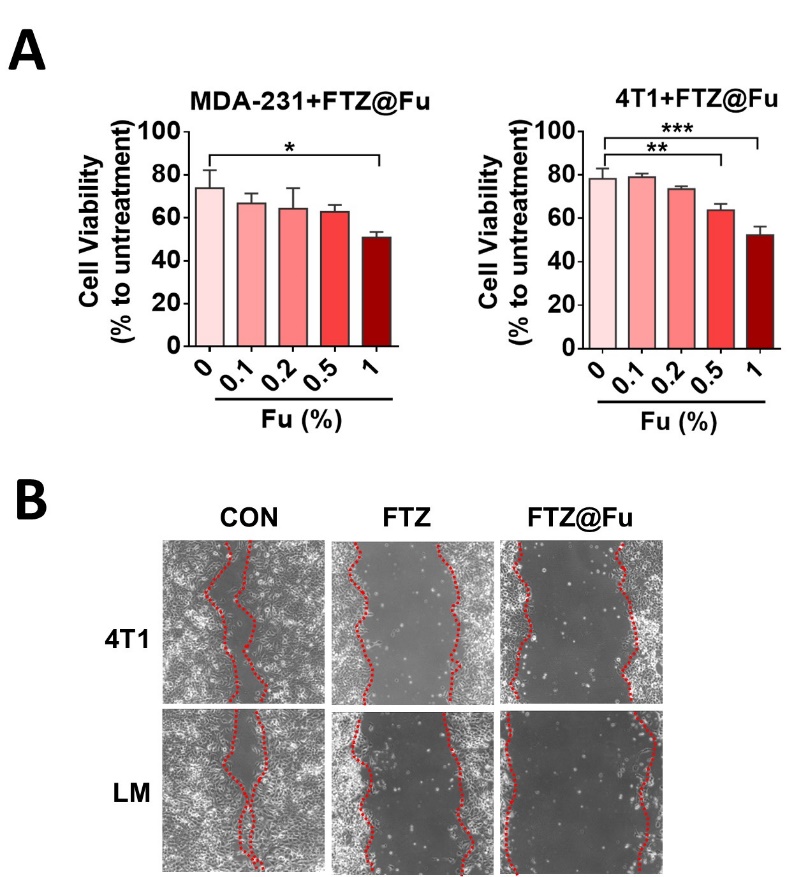
**

**Figure S1 Effect of FTZ SANs on wound healing migration.** (A) Relative cellular viability of breast tumor cells treated with FTZ SANs (5 μg/ml) coated with different concentration of fucoidan. (B)4T1 and LM cells were treated with FTZ and FTZ@Fu (10 μg/ml) for 24 h and photographed by an inverted microscope.


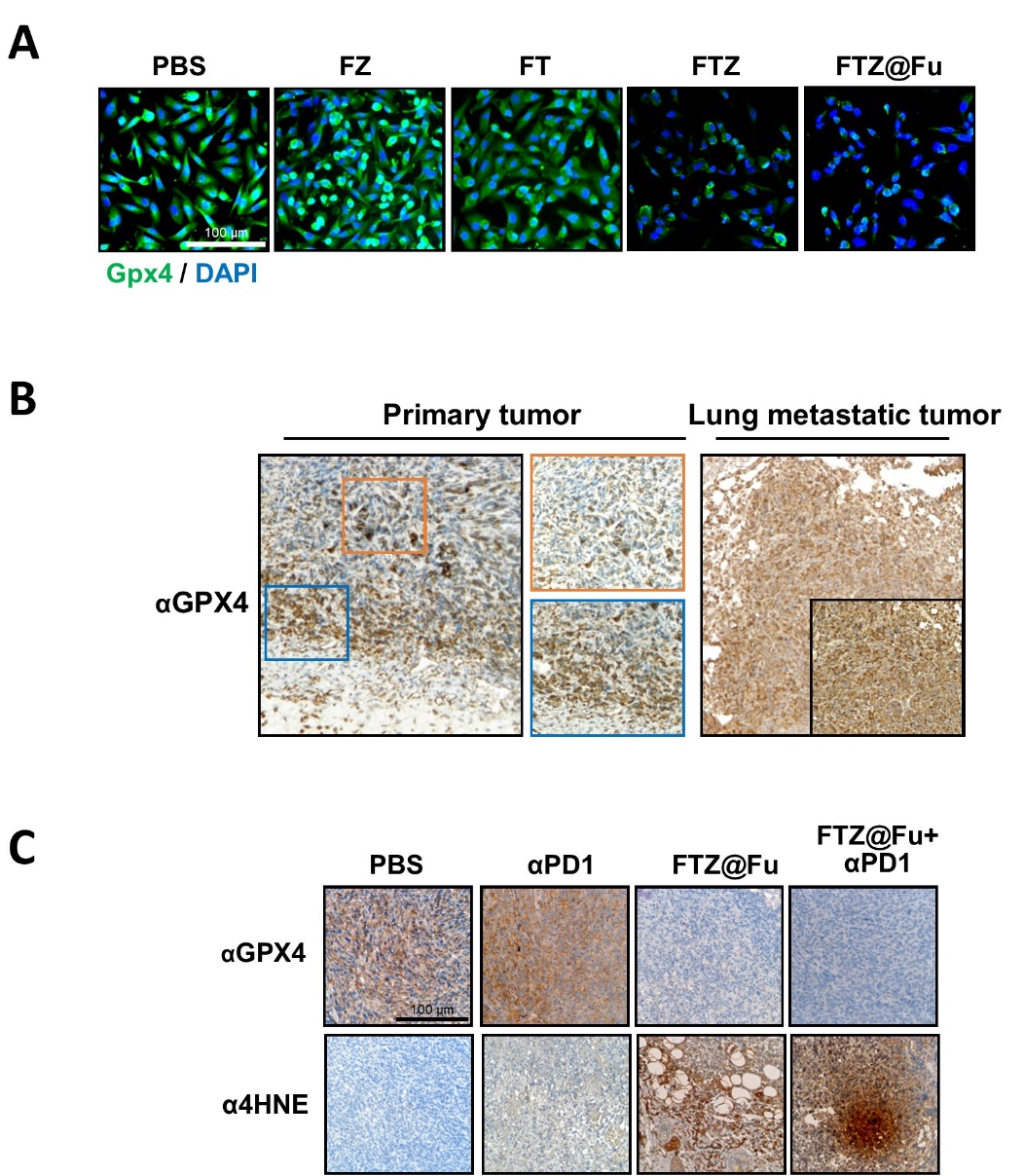


**Figure S2 GPX4 protein level in breast tumor cell and tumor tissues.** (A) Immunofluorescent images of GPX4 in MDA-MB231 cells treated with indicated NPs. (B) Immunohistochemistry analysis of GPX4 in primary and lung metastatic 4T1 tumor tissues. (C) IHC for GPX4 and 4HNE in tumor tissues.


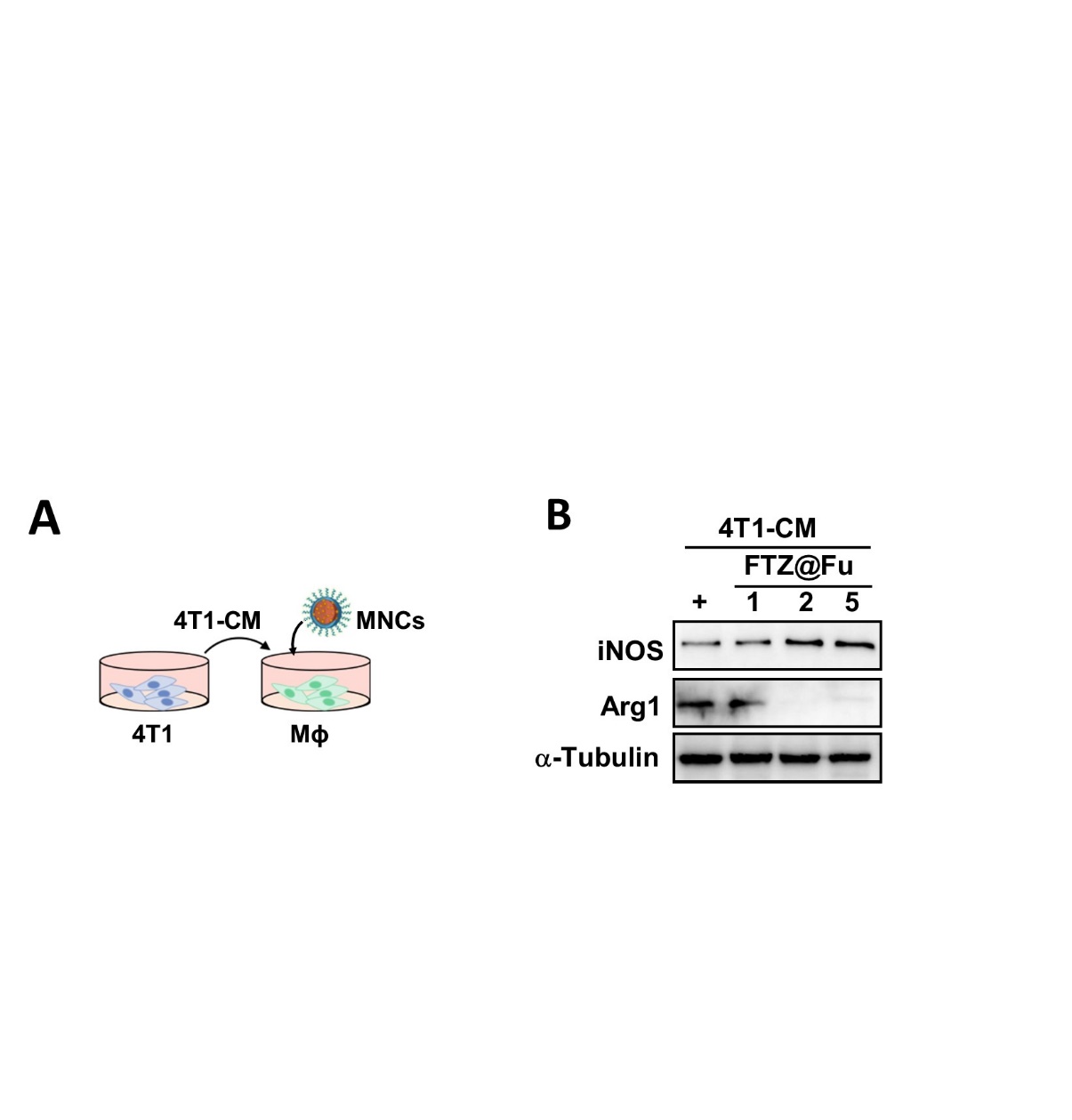


**Figure S3 Effect of FTZ@Fu SANs on macrophage activation.** (A) RAW264.7 macrophages were treated with 4T1-CM in the presence of FTZ@Fu (1-5 μg/ml) for 24 h, and M1 marker (iNOS) and M2 marker (Arginase 1) were examined by Western blot.


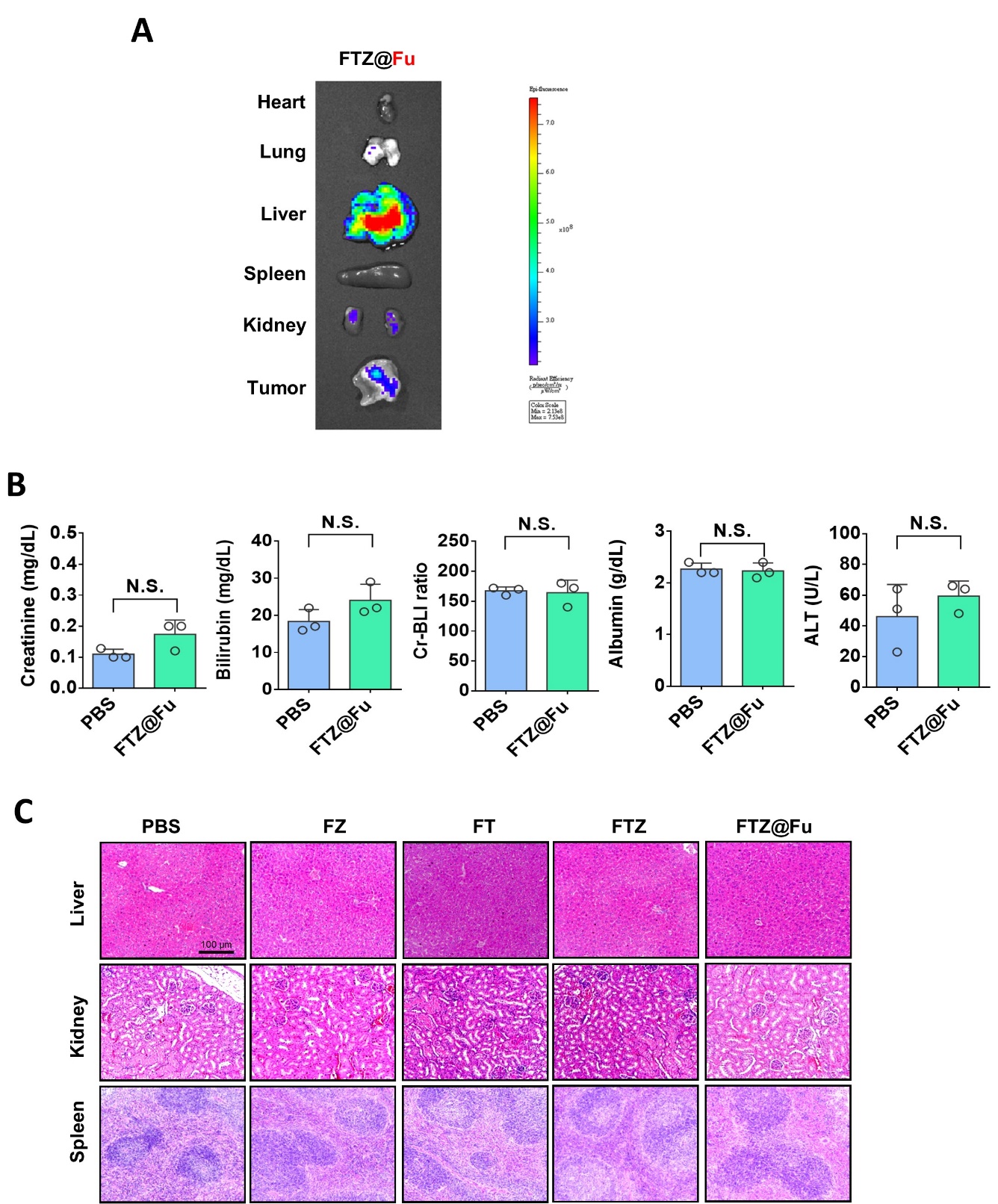


**Figure S4 Biosafety of FTZ@Fu SANs.** (A) *Ex-vivo* fluorescence imaging of various organs and tumors at 24 h after intravenous administration of rhodamine-labeled fucoidan on FTZ@Fu. (B) Serum levels of Cr, BUN, Albumin, and ALT analyses (n = 3/group), N.S. not significant. (C) Representative histological images of organs from BALB/c mice treated with different MNCs formulations. Bar=100 μm.


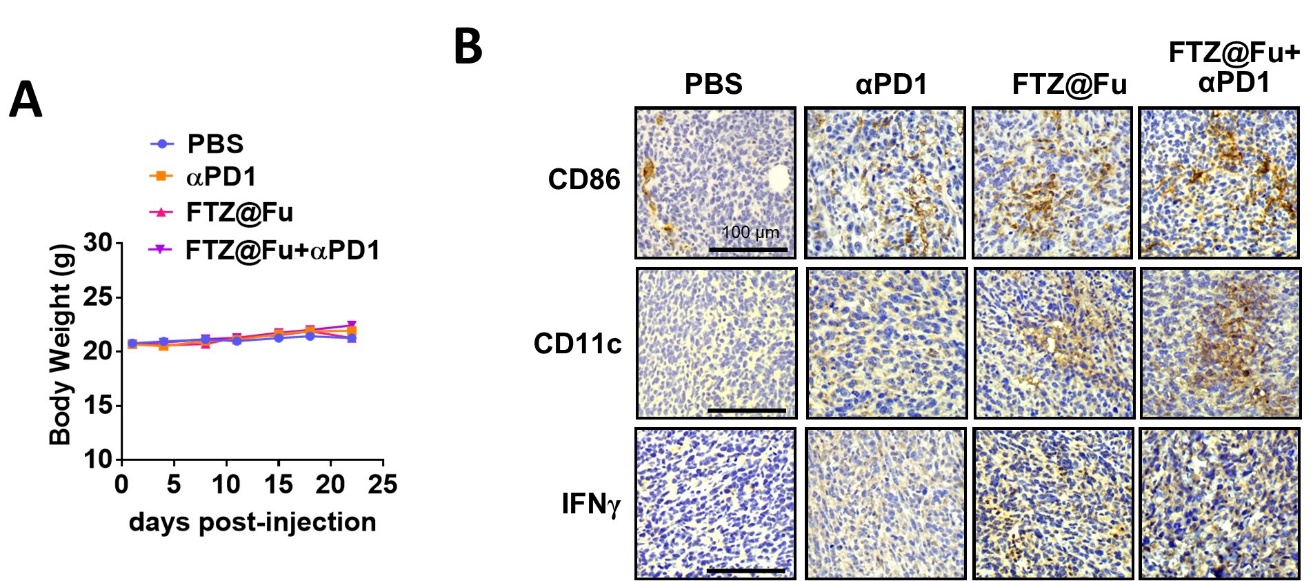


**Figure S5 Effect of FTZ@Fu SANs combined with ICI therapy.** (A) Mice body weight change. (B) IHC images of CD86, CD11c, and IFNγ in tumor tissues. Bar= 100 μm.
